# The role of microglial/macrophagic salt-inducible kinase 3 on normal and excessive phagocytosis after transient focal cerebral ischemia

**DOI:** 10.1007/s00018-022-04465-1

**Published:** 2022-07-21

**Authors:** Ke Wang, Chenran Wang, Di Chen, Yichen Huang, Jiaying Li, Pengju Wei, Ziyu Shi, Yue Zhang, Yanqin Gao

**Affiliations:** State Key Laboratory of Medical Neurobiology, MOE Frontier Center for Brain Science, Institutes of Brain Science, Fudan University, Shanghai, 200032 China

**Keywords:** SIK3, Microglia/macrophages heterogenization, Excessive phagocytosis, Myelin, White matter, Single-cell RNA-seq

## Abstract

Previous studies suggested that anti-inflammatory microglia/macrophages (Mi/MΦ) play a role in “normal phagocytosis,” which promoted the rapid clearance of necrotic substances and apoptotic cells. More recently, a few studies have found that Mi/MΦ also play a role in “pathological phagocytosis” in the form of excessive or reduced phagocytosis, thereby worsening damage induced by CNS diseases. However, the underlying mechanisms and the Mi/MΦ subtypes related to this pathological phagocytosis are still unknown. Salt-inducible kinase 3 (SIK3), a member of the 5’ adenosine monophosphate-activated protein kinase (AMPK) family, has been shown to regulate inflammation in several peripheral diseases. Whether SIK3 also regulates the inflammatory response in CNS diseases is currently unknown. Therefore, in this study, we created a transgenic tamoxifen-induced Mi/MΦ-specific SIK3 conditional knockout (SIK3-cKO) mouse to examine SIK3’s role in phagocytotic function induced by transient focal cerebral ischemia (tFCI). By single-cell RNA-seq, we found the pro-inflammatory Mi/MΦ phenotype performed an excessive phagocytotic function, but the anti-inflammatory Mi/MΦ phenotype performed a normal phagocytotic function. We found that SIK3-cKO caused Mi/MΦ heterogenization from the transitional phenotype to the anti-inflammatory phenotype after tFCI. This phenotypic shift corresponded with enhanced phagocytosis of both apoptotic and live neurons. Interestingly, SIK3-cKO enhanced normal phagocytosis of myelin debris but attenuating excessive phagocytosis of non-damaged myelin sheath, thereby protecting white matter integrity after tFCI. CD16, a pro-inflammation marker, was decreased significantly by SIK3-cKO and correlated with “excessive phagocytosis.” SIK3-cKO promoted long-term recovery of white matter function and neurological function as assessed with electrophysiological compound action potential (CAPs) and behavioral analysis. This study is the first to show a role of SIK3 in Mi/MΦ phagocytosis in CNS diseases, and reveals that promoting Mi/MΦ anti-inflammatory heterogenization inhibits “excessive phagocytosis” of live cells and facilitates “normal phagocytosis” of apoptotic cells. Therefore, inhibition of SIK3 in Mi/MΦ may be a potential therapeutic target in stroke and other CNS diseases with accompanying white matter destruction.

**Graphical abstract:**

In the acute stage of tFCI, Mi/MΦ polarized into different phenotypes. The pro-inflammatory Mi/MΦ phenotype performed an excessive phagocytotic function. In contrast, the anti-inflammatory Mi/MΦ phenotype performed a normal phagocytotic function. After tFCI, SIK3-cKO promoted anti-inflammatory phenotypic heterogenization of Mi/MΦ. SIK3-cKO promoted Mi/MΦ phagocytosis of apoptotic (normal phagocytosis) and living neuronal cell bodies (excessive phagocytosis) in gray matter. Interestingly, SIK3-cKO specifically increased normal phagocytosis of myelin debris concurrent with an attenuation of excessive phagocytosis of myelin sheath in white matter. These changes induced by SIK3-cKO were associated with protection of white matter integrity and long-term neurofunctional recovery after tFCI.

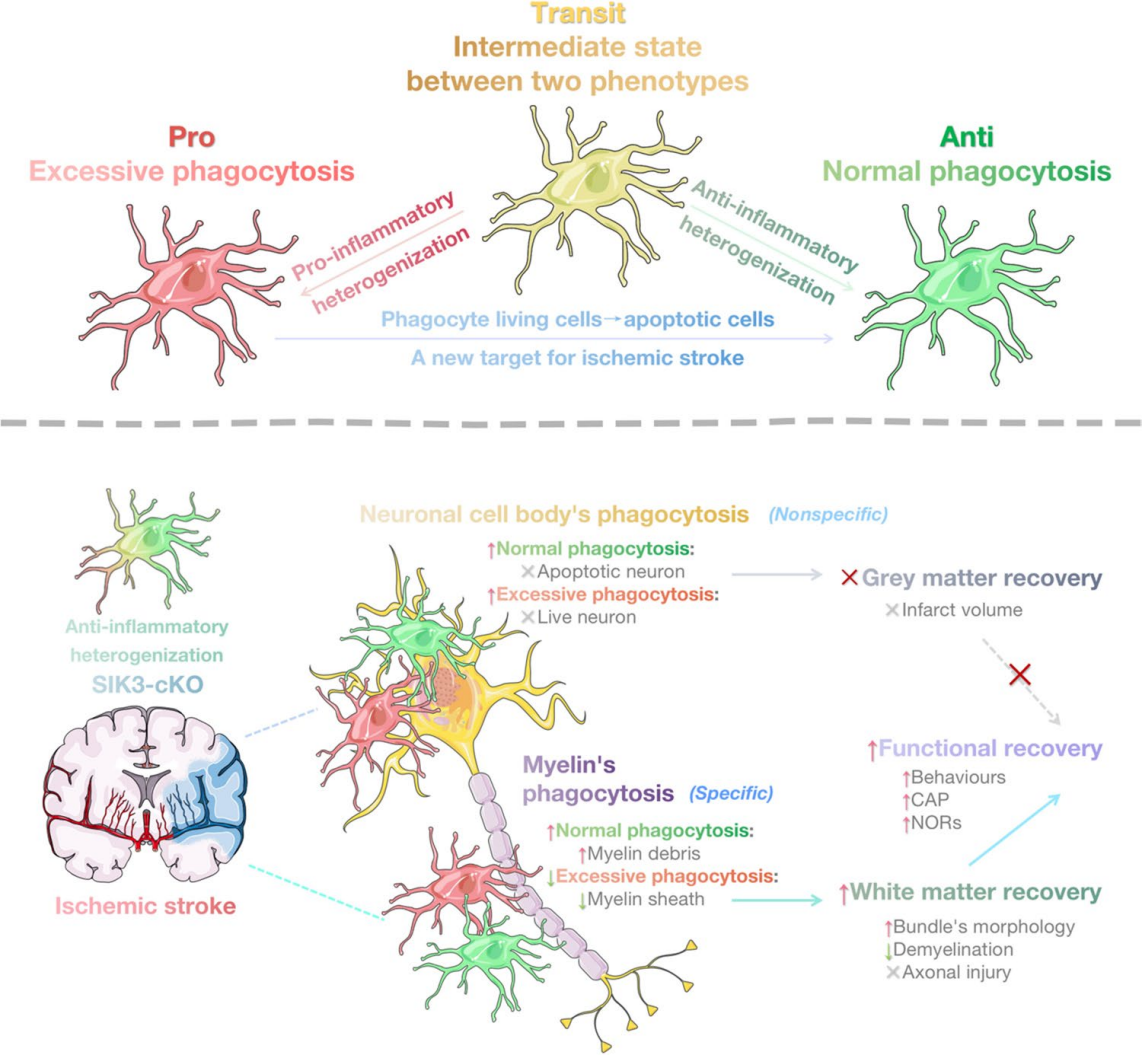

**Supplementary Information:**

The online version contains supplementary material available at 10.1007/s00018-022-04465-1.

## Introduction

Ischemic stroke causes irreversible neurological damage that seriously endangers human health, and is characterized by high morbidity, disability, and mortality. Numerous studies show that intrinsic microglia and peripheral migrating macrophages in the brain rapidly activate and accumulate in the peri-infarct area after stroke [1–3]. The pro-inflammatory phenotype of microglia/macrophages (Mi/MΦ) releases inflammatory factors and recruits inflammatory cells, which in turn exacerbate gray and white matter injuries [4]. In contrast, the anti-inflammatory phenotype of Mi/MΦ improves stroke outcome by promoting phagocytosis of cellular debris, necrotic tissue, and apoptotic cells, resulting in a reduction of toxic and inflammatory substances [5, 6]. As seen with stroke, a significant increase in the phagocytosis of apoptotic neurons was observed with Mi/MΦ heterogenization toward the anti-inflammatory phenotype [7] after traumatic brain injury (TBI). So many studies mistakenly suggested that only the anti-inflammatory Mi/MΦ performed phagocytic functions, and incorrectly believed that all phagocytosis was beneficial. However, recent studies have found that Mi/MΦ not only perform beneficial phagocytosis—“normal phagocytosis” that limited cellular damage, but could also exacerbate cellular damage by way of “pathological phagocytosis.” “Pathological phagocytosis” included excessive or reduced phagocytosis of neuronal cell bodies, synapses, and myelin sheaths, which could be detrimental to histological and functional recovery after stroke [8]. For example, excessive phagocytosis of live neuronal cytosomes by Mi/MΦ contributed to the loss of dopaminergic neurons in Parkinson's disease, which in turn led to abnormal connectivity and function [9, 10]. In chronic ischemia, Mi/MΦ exacerbated white matter injury by phagocyting non-damaged myelin sheath in striatum [11]. However, it remains unclear which Mi/MΦ phenotype is responsible for normal or excessive phagocytosis after stroke. There is little knowledge about the mediator(s) of excessive phagocytosis.

Salt-inducible kinases (SIKs) are members of the AMP-activated protein kinase (adenosine 5'-monophosphate-activated protein kinase, AMPK) family [12]. The SIK family has three isoforms. SIK1 is mainly expressed in the adrenal cortex and SIK2 is expressed in adipose and neural tissues. SIK3 is widely expressed in all tissues of the body [13]. Previous reports related to SIK3 mainly focused on its role in lipid metabolism, bone formation and development, and in mediating inflammation-related effects in the peripheral system [13–16]. Nevertheless, the role of SIK3 in the central nervous system (CNS) has not been widely studied. Several studies have shown that inhibition of SIK3 in macrophages leads to the production of anti-inflammatory factors, such as interleukin (IL)-10, via the CREB-regulated transcription coactivator 3/cAMP-response element binding protein (CRTC3/CREB) pathway in vitro, which results in heterogenization to the anti-inflammatory phenotype [17, 18]. In glial cells in Drosophila, SIK3 regulates potassium and water homeostasis via histone deacetylase 4 [19]. However, whether and how SIK3 contributes to Mi/MΦ phagocytosis remain unknown.

Although it is widely believed that only the anti-inflammatory Mi/MΦ phenotype act as phagocytes, in this study, we found that pro-inflammatory Mi/MΦ also participated in phagocytosis, especially excessive phagocytosis. We discovered that SIK3-Mi/MΦ-specific knockout transgenic mice (SIK3-cKO) shifted Mi/MΦ heterogenization toward the anti-inflammatory phenotype after tFCI. SIK3-cKO promoted non-specific Mi/MΦ phagocytosis of neurons, as both apoptotic and live neurons were phagocyted. In contrast, SIK3-cKO specifically increased normal phagocytosis of myelin debris and decreased excessive phagocytosis of myelin sheath. Phagocytosis resulting from SIK3-cKO functioned to maintain long-term white matter integrity by inhibiting demyelination and promoted better neurofunctional outcomes after tFCI. Therefore, inhibition of SIK3 in Mi/MΦ may be a potential therapeutic target in ischemic stroke and other neurological conditions associated with white matter destruction.

## Results

### The pro-inflammatory Mi/MΦ phenotype tended to perform excessive phagocytosis, whereas the anti-inflammatory Mi/MΦ phenotype tended to perform normal phagocytosis

**Fig. 1:**
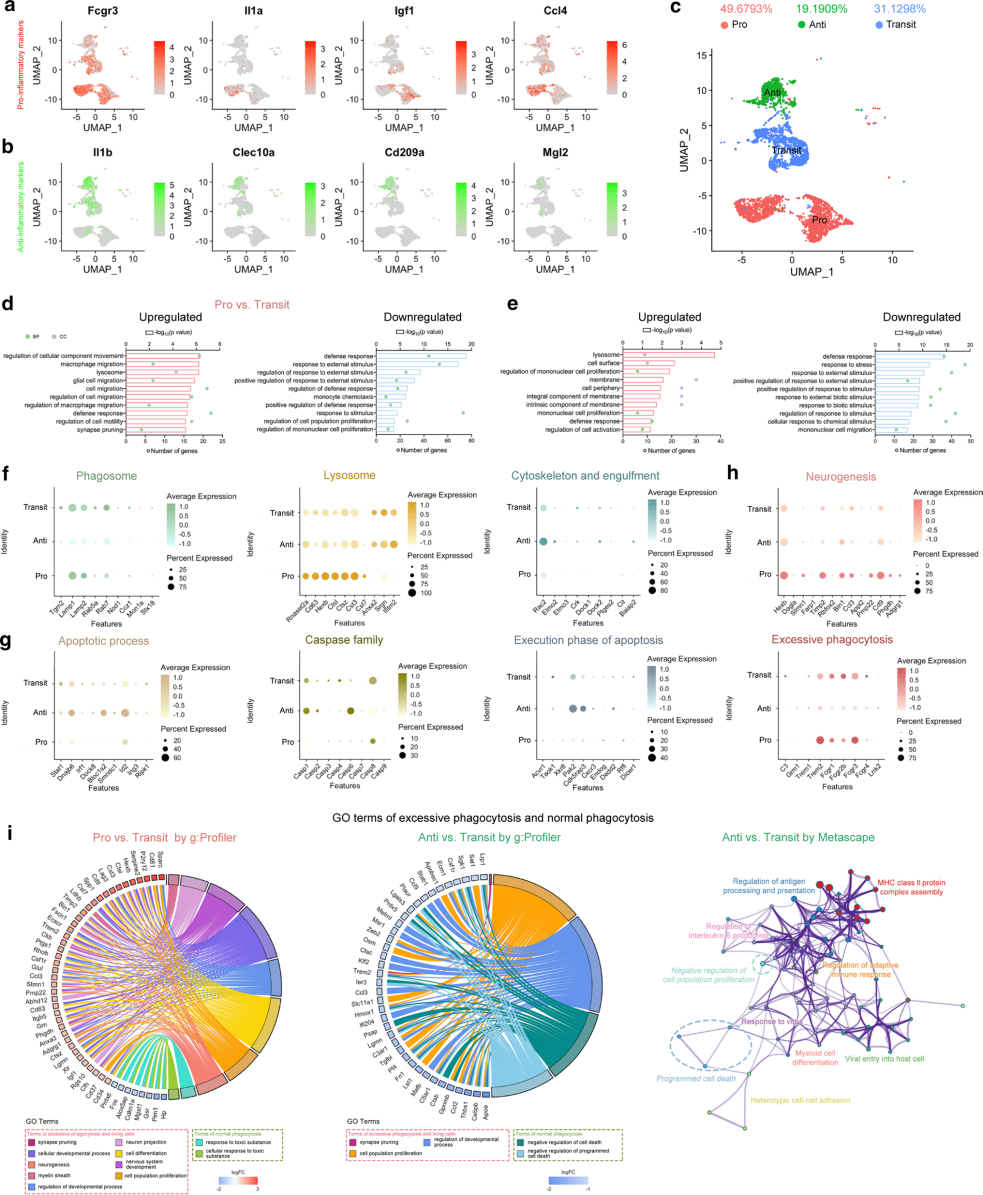
ScRNA-seq after tFCI revealed contrasting phagocytotic functions between the Pro and Anti Mi/MΦ. **a**, **b** Feature plots of pro-inflammatory markers (**a**) and anti-inflammatory markers (**b**) in different clusters of Mi/MΦ. **c** UMAP of the Pro, Anti, and Transit phenotypic Mi/MΦ. **d**, **e** Top 10 GO terms according to − log_10_ FDR of the Pro vs. the Transit phenotype (**d**) and the Anti vs. the transit phonotype (**e**). Enriched GO terms were calculated by g: Profiler. **f**–**h** Bubble matrix of “phagosome,” “lysosome,” “cytoskeleton and engulfment” (**f**), “apoptotic process,” “caspase family,” “execution phase of apoptotic family” (**g**), “neurogenesis,” and “excessive phagocytosis”-related genes in the Pro, Anti, and Transit phenotypic Mi/MΦ. **i** Chord plot and Metascape enrichment map showing GO terms of Mi/MΦ excessive phagocytosis and normal phagocytosis. Left panel: Chord plot showing GO terms of the Pro vs. the Transit phenotype by g: Profiler. Middle panel: Chord plot showing downregulated GO terms of the Anti vs. the Transit phenotype by g: Profiler. Right panel: Enrichment plot showing upregulated GO terms of the Anti vs. Transit phenotype by Metascape

Using published scRNA-seq data (GSM5220257, GSM5220258) of CD45^high^ cells from ischemic mouse brains 5d after 60 min tFCI [20], we classified 10 Mi/MΦ clusters (sFig. 1a, b) into the pro-inflammatory Mi/MΦ (Pro, only expressing the pro-inflammatory markers, including cluster Mi/MΦ1, 2, 6, 8 in the sFig. 1b), the anti-inflammatory Mi/MΦ (Anti, only expressing the anti-inflammatory markers, including cluster Mi/MΦ3, 9, 10 in the sFig. 1b), and the transitional Mi/MΦ (Transit, expressing both pro- and anti-inflammatory markers, including cluster Mi/MΦ4, 5, 7 in the sFig. 1b) polarized phenotypes based on the expression of inflammatory markers (Fig. 1a–c, sFig. 1e–g). The transitional Mi/MΦ could mainly transit into two different directions, the Pro- and Anti- phenotype (sFig. 2a). These clusters of three Mi/MΦ phenotypes were well distinguished by their expression of characteristic genes (sFig. 1e–g). Cx3cr1^CreER^-SIK3^flox/flox^ knockout mice express an enhanced yellow fluorescent protein (eYFP) in Cx3cr1-expressing Mi/MΦ. The flow cytometry was done to analyze the different immunocytes in the brain and blood of Cx3cr1-eYFP mice 3d after tFCI. It showed that the expression level of Cx3cr1 was higher in CD45^+^ Mi/MΦ than other immunocytes (sFig. 1c). Besides, Mi/MΦ markers, such as CD68, Ctsb, and Itgam in CD45 high cells from RNA-seq datasets, also showed that Cx3cr1 and other Mi/MΦ markers are especially expressed in Mi/MΦ after tFCI (sFig. 1d).

A large number of differentially expressed genes (DEGs; fold change > 2 or < -2, adjusted *p* value < 0.05) were identified in the Pro versus the Transit Mi/MΦ and/or in the Anti versus the Transit Mi/MΦ (sFig. 2b). Gene Ontology (GO) enrichment analysis of DEGs was performed by *g: Profiler website*. Hundreds of upregulated and downregulated GO terms were enriched (sFig. 2c). Different GO terms indicated diverse phagocytic functions between the Pro and Anti Mi/MΦ. Consistently, both Pro and Anti Mi/MΦ upregulated genes related to certain phagocytic functions, such as those associated with lysosome and other phagocytic cellular components. In addition, both Pro and Anti Mi/MΦ downregulated genes associated with phagocytic processes in response to certain stimulus. However, the Pro Mi/MΦ had stronger migration abilities than the Anti Mi/MΦ, whereas the Anti Mi/MΦ had more intense proliferation functions than the Pro Mi/MΦ (Fig. 1d). Then, we investigated in more details the phagocytic functions of the Pro, Anti, and Transit Mi/MΦ phenotypes. For phagosome formation, there was higher expression of genes related to the Pro than the Anti phenotype, including lysosome-associated membrane proteins (LAMPs), which are essential to Mi/MΦ phagosome’s maturity and exposure [21, 22], and Rab proteins, which jointly promote phagosome function with Lrrk2 [21]. Lysosomes are an important component of Mi/MΦ ability to digest various substances, and numerous genes related to lysosomes. For example, Hexb and Ctsl were highly activated, especially in the Pro Mi/MΦ phenotype. The Hexb enzyme is essential for Mi/MΦ lysosomal formalization during brain development [23] and Ctsl is a lysosomal protease that degrades cellular proteins via the endolysosomal pathway [24]. Cytoskeleton reformation is a precondition for Mi/MΦ phagocytosis of apoptosis cells [25] and Rac2, which plays a significant role in myotube migration [26], was highly expressed in the Anti Mi/MΦ. Dock2, which mediates cell adhesion and migration by activating Rac and regulates actin cytoskeleton remodeling [26], was also more expressed in the Anti versus the Pro Mi/MΦ phenotype (Fig. 1f). Bridge molecules, “find-me,” “eat-me,” and “don’t eat-me” signals were also differentially expressed in the three clusters (sFig. 2e–g). Notably, contrary to previous reports that only the Anti Mi/MΦ phenotype expressed genes related to phagocytosis [27], we firstly showed that the Pro Mi/MΦ phenotype also possessed these genes and had strong phagocytic function that may be different from the Anti Mi/MΦ.

Mi/MΦ phagocytosis of apoptotic cells or “normal phagocytosis” is beneficial, whereas phagocytosis of living cells or “excessive phagocytosis” [8] can be detrimental. To determine the nature of the phagocytosis of each Mi/MΦ phenotype, we examined the expression of apoptosis-related genes versus genes related to living cells present in each Mi/MΦ phenotype. Dnajb6 was highly expressed in the Anti Mi/MΦ and is purported to mediate apoptosis via the mitochondrial pathway [28]. Caspase kinases, an important apoptosis-related family, were also, highly expressed in the Anti Mi/MΦ phenotype, especially Caspase-1 and Caspase-6. For the execution phase of apoptosis, expression of P21 activated kinases 2 (Pak2), Cdk5rap3, Cxcr3, and Dedd2 was assessed, and was observed to be higher in the Anti phenotype (Fig. 1g). On the other hand, a large number of genes related to neurogenesis, which we used to represent living cells, were highly expressed in the Pro phenotype. For example, Timp2, which plays a role in the withdrawal of progenitor cells from the cell cycle permitting their terminal neuronal differentiation [29], was expressed more in the Pro than the Anti Mi/MΦ phenotype, as were Hexb, Stmn1, Bin1, and Cd9. Signaling molecules previously shown to be involved in pathological phagocytosis [8], such as Trem and Fcgrs (Fig. 1h), were also more highly expressed in the Pro Mi/MΦ phenotype. GO term analysis confirmed that the Pro phenotype upregulated genes related to excessive phagocytosis of living cells, such as myelin sheath, and genes related to regulation of developmental processes and cell proliferation. Terms related to the response to toxic substances were downregulated in the Pro Mi/MΦ. In contrast, GO terms related to excessive phagocytosis were downregulated in the Anti phenotype, including terms related to regulation of developmental process and cell proliferation. Terms related to negative regulation of normal phagocytosis were also downregulated in the Anti phenotype, whereas GO terms related to programmed cell death and negative regulation of cell population proliferation were significantly enriched in the Metascape map (Fig. 1i). Therefore, we surmised that the Anti Mi/MΦ phenotype phagocyted more apoptotic cells and the Pro Mi/MΦ phenotype phagocyted more living cells. And the Pro phenotype Mi/MΦ expressed more genes related to excessive phagocytosis, whereas the anti-inflammatory Mi/MΦ phenotype expressed more genes related to normal phagocytosis, which phagocyted apoptosis cells. According to these results, we inferred that the genes regulating inflammation could promote Mi/MΦ toward anti-inflammatory heterogenization and could thus transform excessive phagocytosis to normal phagocytosis. Importantly genes involved in this heterogenization shift could provide new therapeutic targets in the treatment of ischemic stroke.

### SIK3 in Mi/MΦ was decreased in SIK3-cKO transgenic mice

SIK3-Mi/MΦ-specific knockout transgenic mice (SIK3-cKO) were generated via the Loxp-creER system. There was no significant difference in body weight between wild-type (WT) and SIK3-cKO mice after tamoxifen injection (sFig. 3b). Mice underwent tFCI and laser Doppler flowmetry was used to verify that cerebral blood flow (CBF) dropped to 25% of baseline (see supplementary material for details of experimental methods). There was no difference in CBF (sFig. 3a) or weight loss (sFig. 3c) between WT and cKO mice after surgery.

Using flow cytometry to sort the Mi/MΦ, we confirmed with qPCR that mRNA levels for SIK3, but not SIK1 and SIK2, were decreased in SIK3-cKO mice (sFig. 4a, b). Immunofluorescence staining showed SIK3 protein levels were significantly downregulated in Mi/MΦ in the corpus callosum (CC), the external capsule (EC), and Striatum (STR), but not in the cortex (CTX) (sFig. 4c–g). Our results suggest that SIK3-cKO effectively reduced SIK3 expression in Mi/MΦ, especially in white matter areas, thus confirming the knockout of SIK3 in Mi/MΦ was successful.

### SIK3-cKO inhibited Mi/MΦ activation and promoted their heterogenization to the anti-inflammatory phenotype after tFCI

**Fig. 2:**
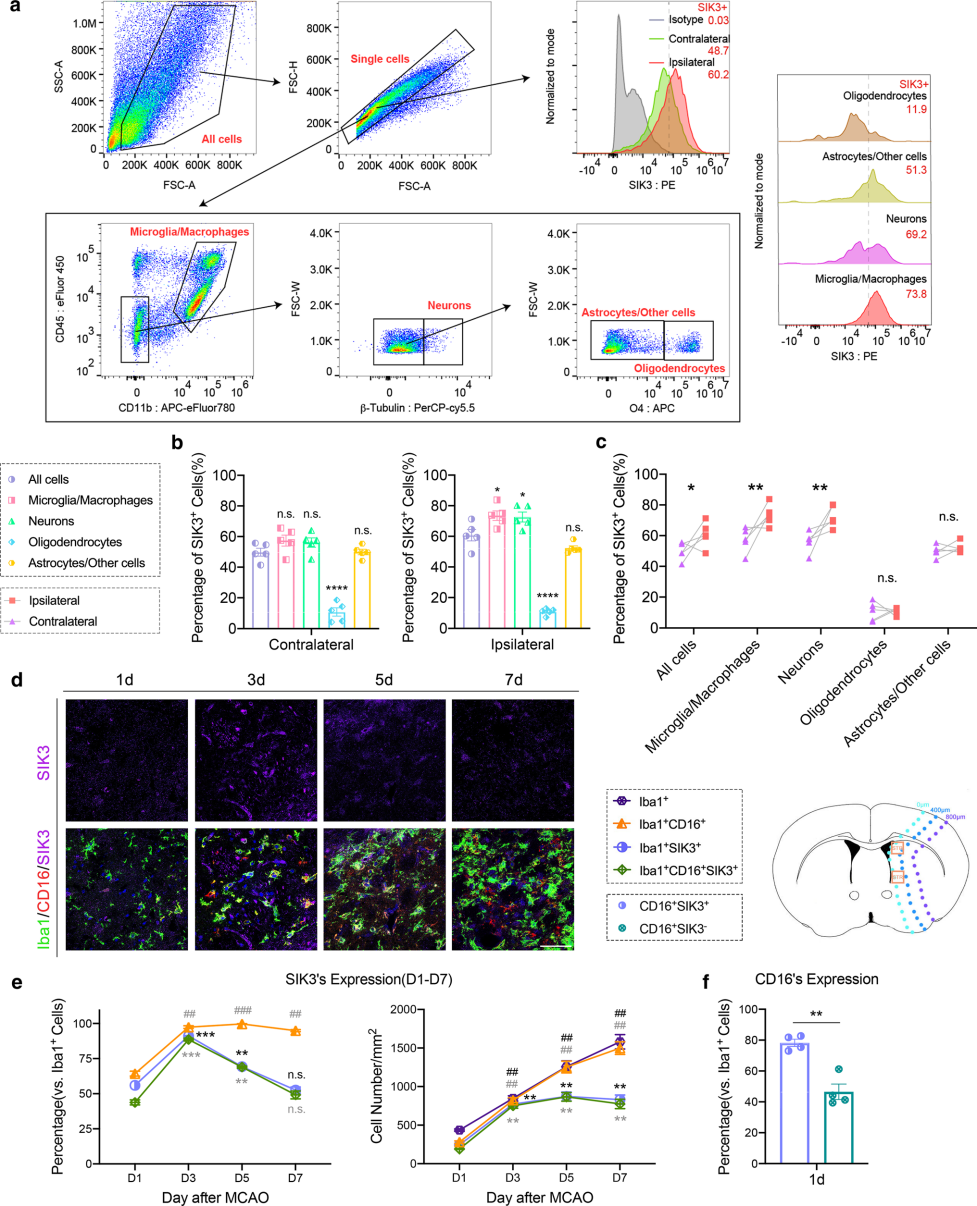
SIK3 is highly expressed in pro-inflammatory Mi/MΦ after tFCI in WT mice. **a** Flow cytometric gating and analysis scheme. **b**, **c** Expression of SIK3 in total cells, Mi/MΦ, neurons, oligodendrocytes, astrocytes and others cells by flow cytometry assessed 3d after tFCI (compared with total groups in the left and middle panel). **d** Representative images of Iba1/CD16/SIK3 triple immunostaining in STR 1, 3, 5, and 7d after tFCI (scale bar: 50 µm). **e** Quantification of the number (right panel) or percentage (left panel) of activated SIK3^+^/CD16^+^ Mi/MΦ in 0–400 µm from the damaged edge (schematic in Fig. 1a down panel) of STR 1, 3, 5, and 7d after tFCI (vs. D1). **f** Quantification of the percentage of CD16^+^ cells in SIK3^+^Mi/MΦ or SIK3^−^Mi/MΦ in 0–400 µm from the damaged edge of STR 1d after tFCI. *N* = 4–5/group. ##*p* < 0.01, ###*p* < 0.001, *p < 0.05, **p < 0.01, ***p < 0.001, ****p < 0.0001, as indicated and assessed by multiple t-tests, one-way ANOVA or two-way ANOVA repeated measurement and Bonferroni post hoc were used for statistical analysis

In WT littermates, tFCI increased the expression of SIK3 in Mi/MΦ and neurons, but not in oligodendrocytes and astrocytes (Fig. 2a–c), which may indicate the special effect of SIK3 in Mi/MΦ after tFCI. We performed ionized calcium binding adaptor molecule 1 (Iba1)/CD16/SIK3 immunofluorescence staining of tissue from WT littermates at 1, 3, 5, and 7d after tFCI to investigate activation and expression of SIK3 in Mi/MΦ after the acute phase of tFCI (Fig. 2d). The results showed that CD16^+^ Mi/MΦ gradually increased over time starting at 3d and remained elevated for at least 7d after tFCI. SIK3 immunofluorescence increased in Mi/MΦ, peaking at day 3 before subsequently decreasing over the next four days (day 7) after tFCI. Interestingly, the temporal dynamics of SIK3^+^CD16^+^ Mi/MΦ were almost similar to the solely SIK3^+^ Mi/MΦ (Fig. 2e). Moreover, the percentage of SIK3^+^CD16^+^ Mi/MΦ was higher (nearly about 80%) than Mi/MΦ not expressing SIK3 (SIK3^−^CD16^+^) 1 day after tFCI (Fig. 2f). Altogether, these results suggest SIK3 may be associated with the pro-inflammatory effects of Mi/MΦ.

**Fig. 3:**
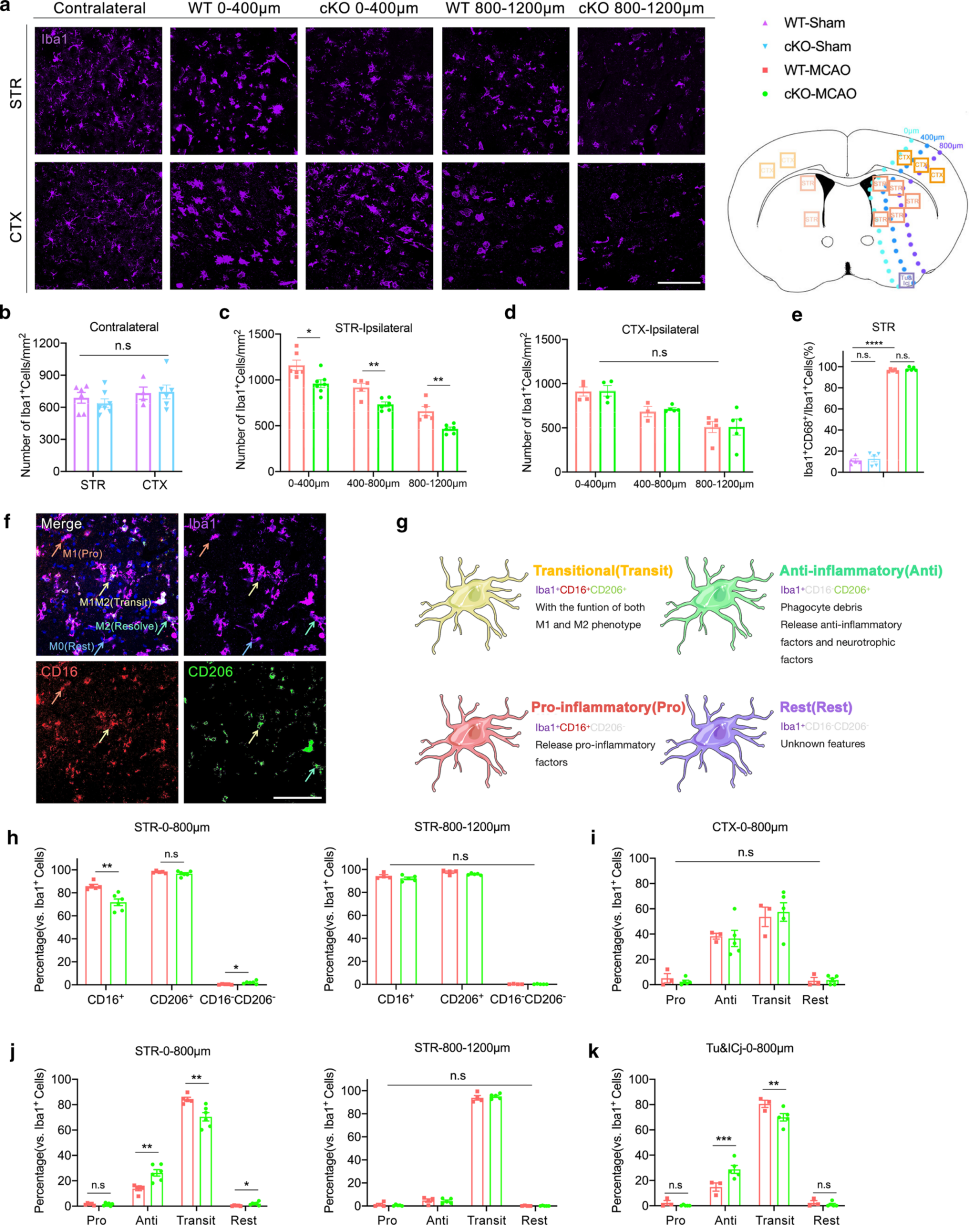
SIK3-cKO inhibited Mi/MΦ activation after tFCI and promoted heterogenization toward the Anti phenotype. **a** Representative images of Iba1 immunostaining in STR and CTX 3d after tFCI (scale bar:100 µm). **b** Quantification of the number of microglia in STR and CTX of the contralateral hemisphere. **c**, **d** Quantification of the number of Mi/MΦ of STR and CTX 3d after tFCI. **e** Quantification of the percentage of CD68^+^ Mi/MΦ in 0–400 µm from the damaged edge in STR 3d after tFCI. **f** Representative images of Iba1/CD16/CD206 triple immunostaining in STR 3d after tFCI (scale bar: 100 µm). **g** Diagrammatic sketch of Mi/MΦ phenotypes. **h** Quantifications of CD16^+^/CD206^+^Mi/MΦ of STR 3d after tFCI. **i**–**k** Quantifications of the Pro, Anti, Transit, and rest phenotype of Mi/MΦ in the penumbra of CTX (i), the core and the penumbra of STR (**j**), and the penumbra of Tu&Icj (**k**) 3d after tFCi. *n* = 3–7/group. *p < 0.05, **p < 0.01, ***p < 0.001, ****p < 0.0001, as indicated and assessed by multiple *t* tests or one-way ANOVA repeated measurement with Bonferroni post hoc analysis

Three days after tFCI (Fig. 3a), there was increased activation and proliferation of Mi/MΦ in WT mice that was greatest in the area 0–400 µm from the ischemic edge, followed by the 400–800 µm area (the ischemic penumbra), and then the 800–1200 µm area (the ischemic core). SIK3-cKO decreased the number of Mi/MΦ in STR (Fig. 3c), but not in CTX (Fig. 3d) relative to WT in all three damaged zones, and did not alter the number of resting microglia in the contralateral hemisphere (Fig. 3b). SIK3-cKO also did not affect the percentage of CD68^+^ activated Mi/MΦ after tFCI as the increase was similar in WT and SIK3-cKO mice (Fig. 3e).

Triple immunofluorescence staining with the pro-inflammatory marker CD16, the anti-inflammatory marker CD206, and Iba1 (Fig. 3f) showed a decrease in CD16^+^ Mi/MΦ concurrent with an increase in CD16^−^CD206^−^Mi/MΦ (Fig. 3h, left panel) in the ischemic penumbra of STR (0–800 µm from the damaged edge) in SIK3-cKO mice. These results demonstrated that SIK3-cKO suppressed the activation of pro-inflammatory Mi/MΦ. However, this heterogenization effect was not observed in the infarct core of STR (800–1200 µm from damaged edge, Fig. 3h, right panel). According to the classification in our previous scRNA-seq analysis, we further classified Mi/MΦ into 4 phenotypes: pro-inflammatory phenotype (Iba1^+^CD16^+^CD206^−^), anti-inflammatory phenotype (Iba1^+^CD16^−^CD206^+^), transitional phenotype (Iba1^+^CD16^+^CD206^+^), and resting phenotype (Iba1^+^CD16^−^CD206^−^). The transit Mi/MΦ express both the pro-inflammatory marker CD16 and the anti-inflammatory marker CD206 (Fig. 3g).

Most Mi/MΦ in the core of STR were of the Transit phenotype and there was no significant difference between WT and SIK3-cKO mice, suggesting that SIK3-cKO did not change the heterogenization of Mi/MΦ in the infarct core (Fig. 3j, right panel). In the penumbra of STR, SIK3-cKO increased the Anti and rest phenotypes, decreased the Transit phenotype, and had no effect on the Pro phenotype (Fig. 3j, left panel). We also observed a similar trend in the olfactory tubercle (Tu&ICj) as in STR (Fig. 3k), but not in the penumbra of CTX (Fig. 3i), which may relate to the fact that SIK3-cKO did not decrease SIK3 expression in CTX after tFCI (sFig. 4e). Taken together, SIK3-cKO suppressed the activation of Mi/MΦ, and induced heterogenization of Mi/MΦ from the Transit phenotype toward the Anti phenotype by reducing CD16^+^ expression in the ischemic penumbra of STR after tFCI.

### SIK3-cKO regulated Mi/MΦ phagocytosis of neuronal cell bodies after tFCI

**Fig. 4:**
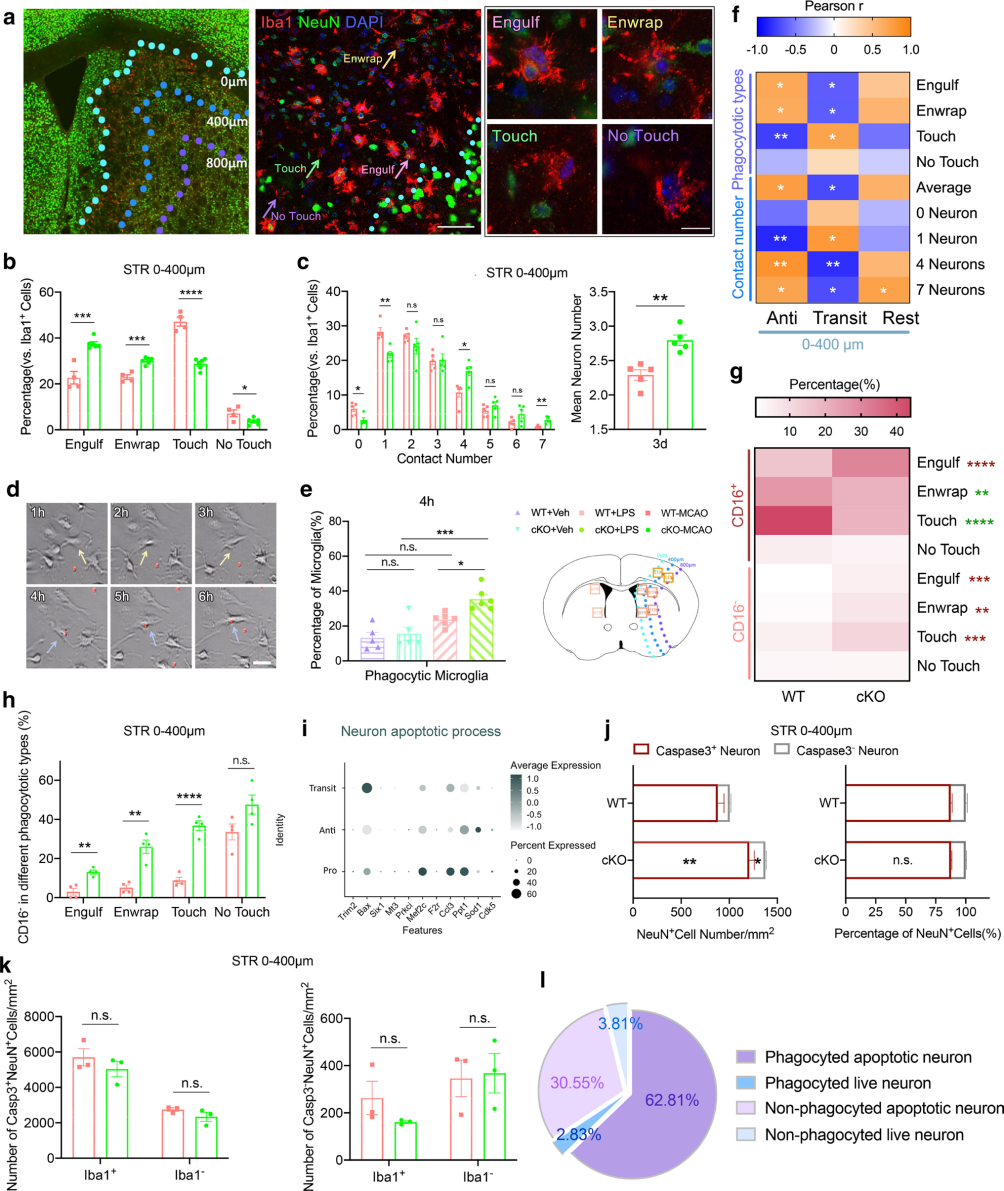
SIK3-cKO promoted Mi/MΦ non-specific phagocytosis of neurons after tFCI. **a** Representative images of Iba1/NeuN double immunostaining in STR 3d after tFCI (middle panel scale bar: 100 µm; right panel scale bar:10 µm). **b** Quantification of the percentage of Mi/MΦ in the “Engulf,” “Enwrap,” “Touch,” and “No Touch” states of phagocytosis in the penumbra within 0–400 µm from the damaged edge in STR 3d after tFCI. **c** Quantification of the percentage of Mi/MΦ that made contact with neurons grouped by the number of contacted neurons in 0–400 µm from the damaged edge of STR 3d after tFCI. **d** Representative images of primary microglia phagocyting 2 µm latex beads 4 h after LPS treatment. Yellow arrows represented non-phagocytic microglia. Blue arrows represented phagocytic microglia (scale bar: 25 µm). **e** Quantification of the percentage of phagocytic Mi/MΦ 4 h after adding latex beads (8 h after LPS treatment). Data are from 3 independent experiments. **f** Correlation analysis of Mi/MΦ with the Anti, Transit, or rest phenotype in the different phagocytic states in 0–400 µm from the damaged edge of STR 3d after tFCI. **g** Correlation of CD16^+^ or CD16^−^ with “Engulf,” “Enwrap,” “Touch” and “No Touch” phagocytic states in 0–400 µm from the damaged edge of STR 3d after tFCI. **h** Quantification of the percentage of CD16^−^ Mi/MΦ in the “Engulf,” “Enwrap,” “Touch” and “No Touch” states, respectively, in 0–400 µm from the damaged edge of STR 3d after tFCI. *n* = 4/group. **i** Bubble matrix of “neuron apoptotic process”-related genes in the pro-, anti-inflammatory, and the transitional Mi/MΦ. **j** Quantification of the number/percentage of Caspase3^+^NeuN^+^ or Caspase3^−^NeuN^+^ in the Mi/MΦ in the “Engulf” state in 0–400 µm from the damaged edge of STR 3d after tFCI. **k** Quantification of the number of apoptotic and live neurons in 0–400 µm from the damaged edge of STR 3d after tFCI. *n* = 3–6/group. **l** Quantification of the percentage of apoptotic and live neurons in WT mice in 0–400 µm from the damaged edge of STR 3d after tFCI. *n* = 3/group. *p < 0.05, **p < 0.01, ***p < 0.001, ****p < 0.0001, as indicated using multiple t tests with Bonferroni post hoc analysis

Activated Mi/MΦ upregulates phagocytic activity and promotes phagocytosis of necrotic tissue, apoptotic cells, and inflammatory toxins [30, 31]. Highly activated Mi/MΦ express lysosome marker-CD68 and display phagocytic behavior. Since there was no difference in the percentage of CD68^+^Mi/MΦ between the WT and SIK3-cKO mice before or after tFCI (Fig. 3e), we examined the nature of the phagocytic behavior of these Mi/MΦ cells (e.g., positive or negative phagocytosis of target cells) in these two groups of mice. During the acute phase after tFCI, activated Mi/MΦ rapidly migrate to the infarct area. They wrap around and digest apoptotic neurons to promote clearance of neuronal debris leading to a better recovery environment for tissue regeneration and functional remodeling [32]. Thus, we performed Iba1/NeuN double-labeled immunostaining (Fig. 4a, middle panel) to investigate how SIK3-cKO regulated Mi/MΦ phagocytosis of neurons. In the contralateral hemisphere, SIK3-cKO had no effect in the normal physiological state. The processes of each resting microglia contacted 2–9 healthy neuronal cell bodies, which on average was 5.5 neuronal cell bodies (sFig. 5a). We divided the degree of phagocytosis of Mi/MΦ into 4 phagocytic states, such as “Engulf,” “Enwrap,” “Touch,” and “No touch” (Fig. 4a, right panel) and ranked them by the number of neuronal contacts (details in the methods section). SIK3-cKO increased Mi/MΦ in the “Engulf” and “Enwrap” phagocytic states and decreased the Mi/MΦ in the “Touch” and “No Touch” phagocytic states in STR (Fig. 4b, sFig. 5b), but not in CTX (sFig. 5d), which is consistent with the effect of SIK3-cKO on Mi/MΦ heterogenization in STR versus CTX (Fig. 3i). After microglia activation under pathological conditions, microglia typically tend to contact fewer neurons due to the enlargement of the cell body and shortening of processes [25]. In the ipsilateral hemisphere, SIK3-cKO significantly decreased the number of Mi/MΦ that contacted 0 and 1 neuron and increased the number of Mi/MΦ that made contact with 4 or 7 neurons. Overall, the average number of neurons contacted by each Mi/MΦ was significantly increased by SIK3-cKO (Fig. 4c). A similar trend was observed in primary microglia after LPS treatment (Fig. 4d, sFig. 3e). SIK3-cKO increased bead-phagocyted microglia after LPS (Fig. 4e, sFig. 5c). These results demonstrate that SIK3-cKO induced Mi/MΦ to contact a greater number and larger area of neurons, which subsequently enhanced the phagocytosis of neurons by these Mi/MΦ.

Since numerous studies have reported that phagocytosis of Mi/MΦ polarized to the Anti phenotype facilitate resolution of the inflammatory response [4, 5, 27, 31], we correlated the Anti, Transit, and rest phenotypes (these were significantly changed by SIK3-cKO, Fig. 3j) with the Mi/MΦ phagocytic state. The results showed the Anti phenotype was positively correlated with those Mi/MΦ increased by SIK3-cKO, which was in the “Engulf,” and “Enwrap” states, and that contacted 4 or 7 neurons. Further, the Anti phenotype was negatively correlated with Mi/MΦ in the “Touch” state and that contacted 1 neuron, which were decreased by SIK3-cKO. In contrast, the opposite changes were observed with SIK3-cKO in Mi/MΦ of the Transit phenotype. Indeed, SIK3-cKO decreased the number of Mi/MΦ of the Transit phenotype in the “Engulf” and “Enwrap” state, as well as those that contacted 4 or 7 neurons, while increasing those in the “Touch” state and that contacted 1 neuron (Fig. 4f). These results suggested that SIK3-cKO increased phagocytic activity by converting Mi/MΦ from the Transit phenotype to the Anti phenotype.

Mi/MΦ perform both positive “normal phagocytosis” and negative “pathological phagocytosis” [8]. Therefore, we investigated whether SIK3-cKO played a role in normal phagocytosis of apoptotic neurons or excessive phagocytosis of live neurons. Since SIK3-cKO reduced CD16 expression in Mi/MΦ (Fig. 3h), we used Iba1/CD16/NeuN triple immunostaining (sFig. 5e) to assess the phagocytosis of neurons by pro-inflammatory Mi/MΦ. We found that both CD16^+^ and CD16^−^ Mi/MΦ showed a phagocytotic morphology of the “Engulf,” “Enwrap,” and “Touch” states. SIK3-cKO enhanced CD16^−^ Mi/MΦ, but inhibited CD16^+^ Mi/MΦ phagocytosis (Fig. 4h–i). Furthermore, SIK3-cKO increased the “Engulf,” “Enwrap,” and “Touch” phagocytic states in CD16^−^ Mi/MΦ. The CD16^+^Enwrap and CD16^+^Touch types were decreased by SIK3-cKO, whereas the number of Mi/MΦ in the CD16^+^Engulf state was increased 3d after tFCI (Fig. 4g). These data suggested SIK3-cKO promoted Mi/MΦ phagocytosis by regulating anti-inflammatory Mi/MΦ.

To determine whether Mi/MΦ selectively phagocyted apoptotic or live neurons, we analyzed genes related to neuron apoptotic process by scRNA-seq. There was no difference in RNA expression of apoptotic neurons in the Pro, Anti, and Transit Mi/MΦ (Fig. 4i). Furthermore, we performed Iba1/NeuN/Caspase3 triple immunostaining (sFig. 5f) and quantified the number of apoptotic or live neurons only in the “Engulf” phagocytic state. Compared to WT mice, SIK3-cKO significantly increased the number of apoptotic neurons (Caspase3^+^NeuN^+^) and live neurons (Caspase3^−^NeuN^+^) that were engulfed without changing the percentage of apoptotic and live neurons engulfed (Fig. 4j). These data indicated SIK3-cKO similarly increased normal Mi/MΦ phagocytosis of apoptotic neurons and excessive phagocytosis of live neurons. Interestingly, there was no difference in the number of apoptotic and live neurons in the 0–400 µm area in STR between WT and SIK3-cKO mice 3d after tFCI (Fig. 4k). In WT mice, Mi/MΦ phagocyted 62.81% apoptotic neurons and approximately 2.83% live neurons (Fig. 4l). Overall, these data confirm that SIK3-cKO promoted the indiscriminate phagocytosis of neurons by Mi/MΦ, as apoptotic neurons as well as live neurons were cleared. Maybe Mi/MΦ could not recognize apoptotic and live neurons after ischemic stroke.

### SIK3-cKO regulated Mi/MΦ phagocytosis of myelin after tFCI

**Fig. 5:**
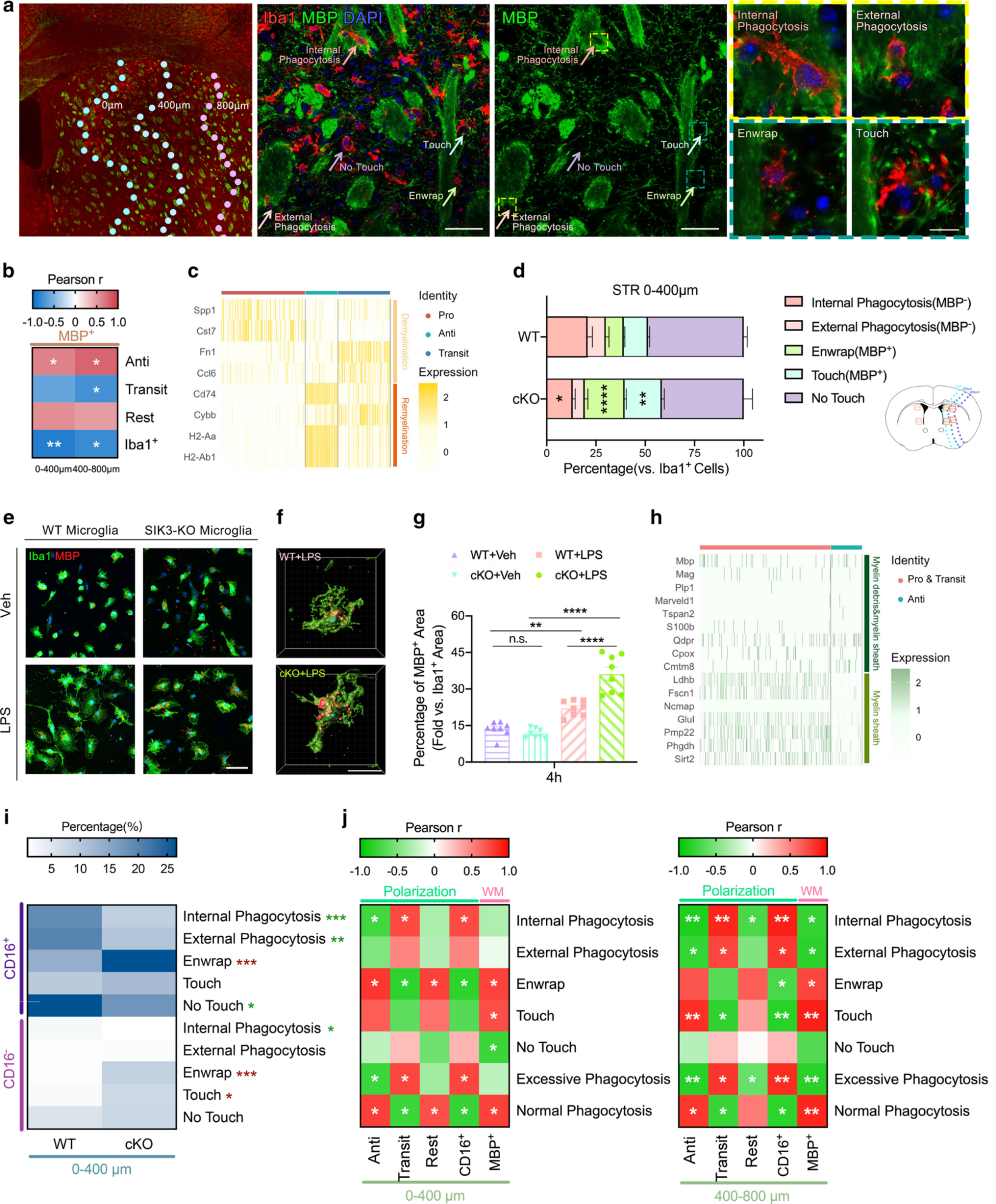
SIK3-cKO inhibited excessive phagocytosis of myelin sheath and promoted normal phagocytosis of myelin debris by inhibiting Mi/MΦ pro-inflammatory heterogenization. **a** Representative images of Iba1/MBP immunostaining (middle panel, scale bar: 100 µm) in STR (left panel) and four typical phagocytic states (right panel, scale bar: 10 µm) 3d after tFCI. **b** Correlation of myelin integrity and Mi/MΦ activation/heterogenization in the penumbra of STR (0–400 µm and 400–800 µm from the damaged edge) 3d after tFCI. **c** Heat map of demyelination- and remyelination-related genes in the pro-, anti-inflammatory, and the transitional phenotypic Mi/MΦ. **d** Quantification of the percentage of “Internal phagocytosis,” “External phagocytosis,” “Enwrap,” “Touch,” and “No Touch” phagocytic states of Mi/MΦ in 0–400 µm of STR from the damaged edge 3d after tFCI. **e** Representative images of Iba1/MBP immunostaining (scale bar: 50 µm) of primary microglia 4 h after adding myelin debris. **f** 3D rendered images of Iba1/MBP immunostaining (scale bar: 20 µm) of primary microglia in WT-LPS and cKO-LPS groups 4 h after adding myelin debris. **g** Quantification of the percentage of MBP^+^ area versus Iba1^+^ area 4 h after adding myelin debris (8 h after LPS treatment). *n* = 8/group. **h** Heat map of “myelin debris and myelin sheath” and “myelin sheath”-related genes in the pro-, anti-inflammatory, and the transitional phenotypic Mi/MΦ. **i** Quantifications of the percentage of CD16^+^/CD16^−^ in 5 phagocytic types of Mi/MΦ in the area 0–400 µm from the damaged edge in the penumbra of STR 3d after tFCI. **j** Correlation analysis of the Anti, Transit, rest phenotypes, percentage of Iba1^+^CD16^+^ and myelin integrity with phagocytic types of Mi/MΦ in the area 0–400 µm and 400–800 µm from the damaged edge of STR 3d after tFCI. *n* = 4–6/group. *p < 0.05, **p < 0.01, ***p < 0.001, ****p < 0.0001, as indicated. Multiple *t* tests and Bonferroni post hoc were used for statistical analysis

Myelin bundles are an important component of white matter. Ischemic stroke causes severe and extensive demyelination [33, 34]. However, most studies only focused on beneficial phagocytosis of myelin debris [35] by Mi/MΦ, but ignored the fact that Mi/MΦ could also induce demyelination [11]. Almost no studies have investigated both the harmful pathological phagocytosis of myelin sheath and beneficial phagocytosis of myelin debris simultaneously in CNS diseases [8]. By correlating myelin integrity and Mi/MΦ phenotypes, we found that myelin integrity was positively correlated with the Anti phenotype and negatively correlated with the Transit phenotype and Mi/MΦ activation (Fig. 5b). By scRNA-seq, demyelination-related genes were highly expressed in the Pro Mi/MΦ, remyelination-related genes were highly expressed in the Anti Mi/MΦ (Fig. 5c) [36]. To examine the nature of Mi/MΦ phagocytosis of myelin after tFCI, we performed Iba1/MBP (myelin basic protein) immunofluorescence staining (Fig. 5a). The nature of the interaction between Mi/MΦ and bundles varied based on whether the phagocytosis was deemed excessive or normal, so we morphologically classified Mi/MΦ phagocytosis of myelin into 5 phagocytic types (details in the methods section): “excessive phagocytosis (myelin loss, MBP^−^)” with (1) “Internal phagocytosis” or (2) “External phagocytosis” morphology, “normal phagocytosis (myelin complete, MBP^+^)” with (3) “Enwrap” or (4) “Touch” morphology, and (5) non-phagocytosis (myelin complete, MBP^+^) with “No Touch” morphology (Fig. 5a, right panel). Results showed SIK3-cKO reduced excessive phagocytosis of both types (“Internal” and “External”), while increasing both types of normal phagocytosis (“Enwrap” and “Touch”) after tFCI (Fig. 5d, sFig. 6a). In lieu of in vivo studies examining the nature of phagocytosis of myelin debris, we used primary microglia treated with LPS to examine this process (sFig. 3e), which could not be seen clearly by in vivo studies. Iba1/MBP double immunofluorescence staining showed SIK3-cKO promoted normal phagocytosis of myelin debris by microglia (Fig. 5e–g, sFig. 6b).

We further investigated how SIK3-cKO alters Mi/MΦ heterogenization to regulate phagocytosis of myelin. Results of scRNA-seq showed pro-inflammatory Mi/MΦ phagocyted more myelin sheath. Besides, markers of myelin and mature oligodendrocytes (myelin sheath and myelin debris included) were higher in anti-inflammatory Mi/MΦ, revealing that there was more myelin debris in anti-inflammatory Mi/MΦ (Fig. 5h). Through triple immunofluorescence staining with Iba1/CD16/MBP (sFig. 6c), we found that CD16^+^ Mi/MΦ phenotype mainly presented in the “Internal,” “External” and “No touch” phagocytic states in WT mice, while CD16 was highly expressed in the “Enwrap” state in the SIK3-cKO mice 3d after tFCI (Fig. 5i). Thus, SIK3-cKO may reduce white matter injury by shifting the behavior of Mi/MΦ from pathological phagocytosis to normal phagocytosis, thereby limiting the damage induced by excessive clearance of viable myelin sheath and promoting beneficial phagocytosis of myelin debris.

We correlated the phagocytic states of Mi/MΦ with MBP intensity and different heterogenization phenotypes to further determine if the shift in the phagocytic states underlay the protection of white matter integrity induced by SIK3-cKO after tFCI. The results showed the normal phagocytic states were positively correlated with the anti-inflammatory Mi/MΦ phenotype and MBP intensity, but negatively correlated with the pro-inflammatory phenotype. In contrast, the excessive phagocytic states were positively correlated with the pro-inflammatory phenotype, but negatively correlated with MBP intensity, especially in 400–800 µm from the damaged edge of STR (Fig. 5j). These data suggested that SIK3-cKO after tFCI inhibited demyelination by converting the function of Mi/MΦ from excessive phagocytosis to normal phagocytosis via heterogenization toward anti-inflammatory phenotype. Notably, unlike the indiscriminate phagocytosis of neurons, Mi/MΦ could specifically recognize myelin debris from myelin sheath.

### SIK3-cKO did not alleviate gray matter injury but alleviated white matter injury by protecting myelin integrity after tFCI

**Fig. 6:**
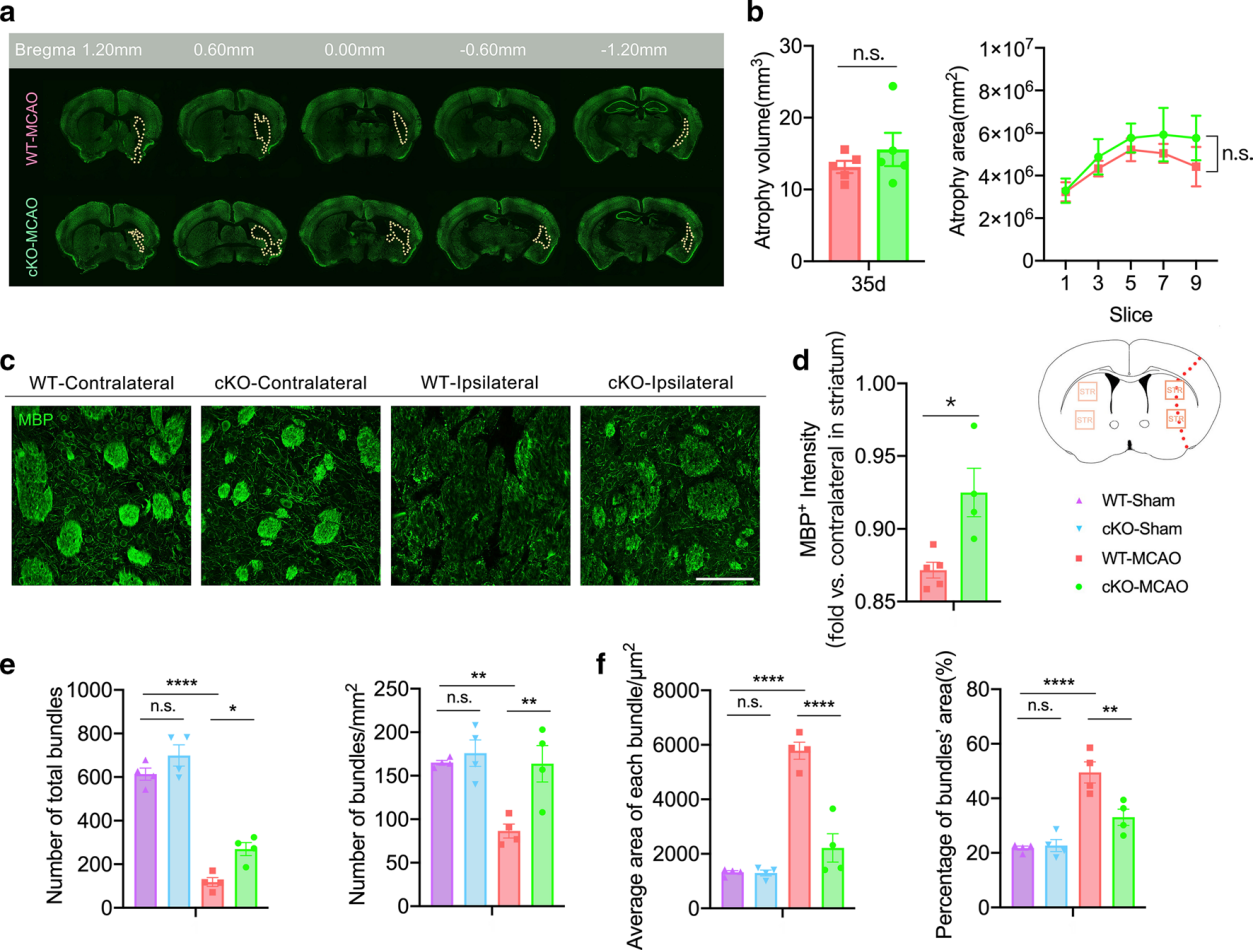
SIK3-cKO alleviated long-term demyelination and bundle atrophy after tFCI. **a** Representative images of NeuN immunostaining 35d after tFCI. **b** Quantification of infarct volume/area 35d after tFCI. *n* = 5/group. **c** Representative images of MBP immunostaining in STR 35d after tFCI (scale bar = 100 µm). **d** Quantification of the MBP density in the penumbra of STR 35d after tFCI. **e** Quantification of the number of fiber bundles in the whole STR 35d after tFCI. **f** Quantification of the average area of each bundle, and the percentage of fiber bundle area in the whole STR 35d after tFCI. *n* = 4–5/group. *p < 0.05, **p < 0.01, ****p < 0.0001. ns: no significant, as indicated. One-way ANOVA or two-way ANOVA or Mutiple t tests were used to assess statistical analysis

Our results suggested SIK3-cKO differentially regulated Mi/MΦ phagocytosis of neurons (gray matter) compared with myelin (white matter). Therefore, we investigated whether this differential regulation influenced the fate of gray and white matter at the histological level. tFCI causes acute and long-term gray matter damage characterized by neuronal apoptosis and loss [37, 38]. NeuN immunostaining 35d after tFCI was performed to assess infarct/atrophy volumes. SIK3-cKO did not reduce atrophy volume in long term (Fig. 6a, b). This result was consistent with data indicating no significant difference in Mi/MΦ phagocytosis of neurons 3d after tFCI.

In addition to gray matter injury, severe and diffuse white matter injury after tFCI is also significantly correlated with patients’ prognosis. White matter injury includes demyelination and axonal injury [34, 39, 40]. tFCI not only led to significant demyelination, but also resulted in severe deformation of structure of the fiber bundle in STR long term [33, 34, 39, 41, 42]. By performing MBP immunostaining 35 days after tFCI (Fig. 6c), we observed that a great amount of myelin was lost in STR of WT mice, while SIK3-cKO protected the integrity of myelin by inhibiting demyelination and/or promoting remyelination (Fig. 6d). tFCI caused a decrease in fiber bundle numbers, but SIK3-cKO attenuated this loss in bundle numbers (Fig. 6e). The bundle area was increased in the coronal brain section due to structure of the fiber bundle being loose after tFCI, while SIK3-cKO facilitated the recovery of bundle morphology (Fig. 6f). In conclusion, SIK3-cKO alleviated white matter injury long term after tFCI by protecting against demyelination and promoting recovery of normal bundle morphology. Thus, the mechanism by which SIK3-cKO inhibited Mi/MΦ excessive phagocytosis to ameliorate demyelination during the acute phase could persist long term after tFCI.

### SIK3-cKO promoted functional recovery after tFCI

**Fig. 7:**
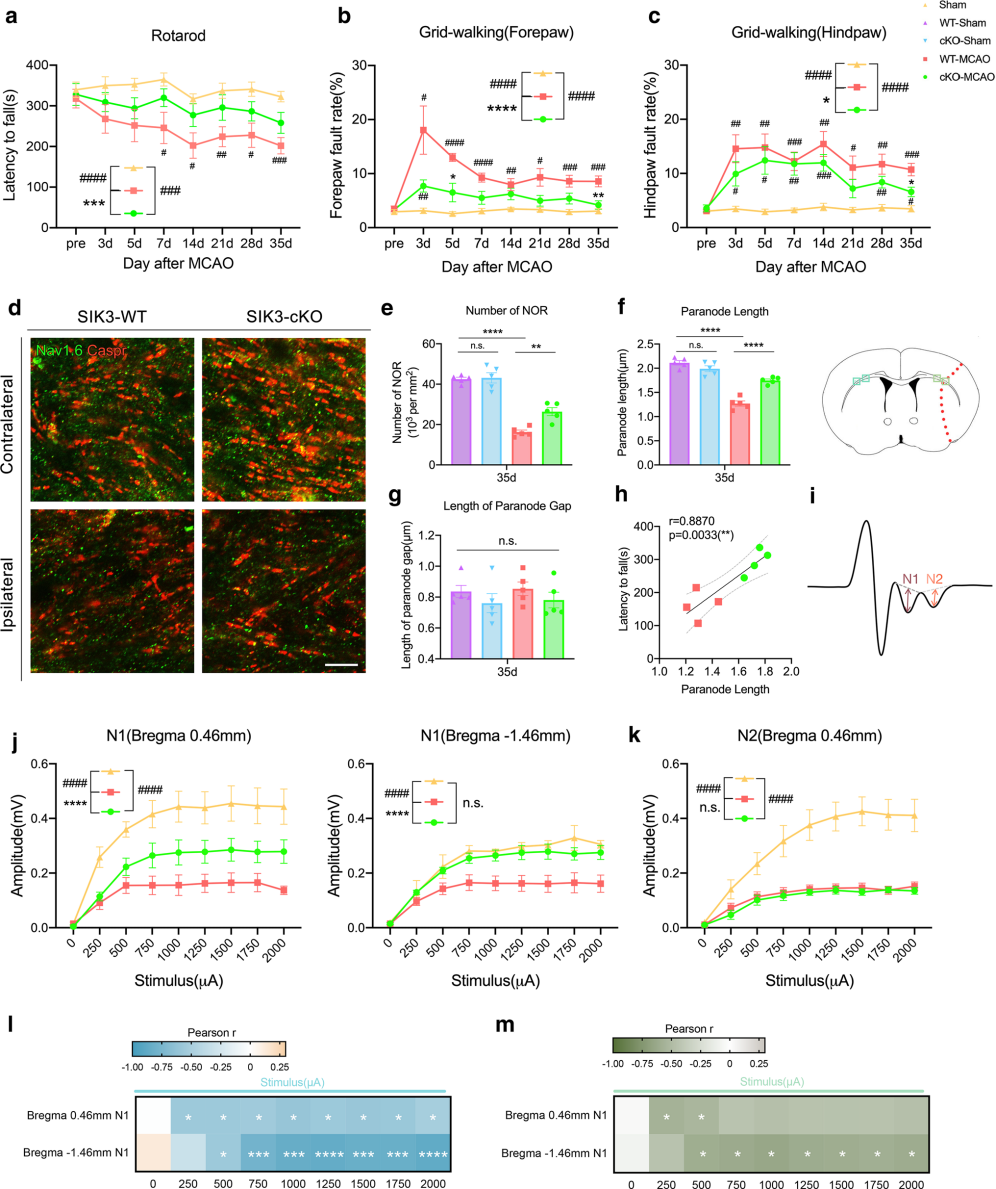
SIK3-cKO promoted long-term functional recovery after tFCI. **a**–**c** Sensorimotor deficits were evaluated with rotarod (**a**) and grid-walking (**b**, **c**) tests up to 35d after tFCI. *n* = 8–11/group. **d** Representative images of Nav1.6/Caspr immunostaining in the external capsule (EC) area 35d after tFCI (scale bar = 5 µm). **e**–**g** Quantification of the number of Ranvier nodes (**e**), length of Ranvier nodes (**f**), and length of paranode gap (**g**) in the EC area 35d after tFCI. *n* = 5/group. **h** Correlation of paranode length and latency to fall 35d after tFCI. *n* = 4/group. **i** Representative images of CAP N1/N2 35d after tFCI. **j** Amplitude of N1 at the level of Bregma + 0.46 mm and Bregma − 1.46 mm 35d after tFCI. *n* = 4–6/group. **k** Amplitude of N2 at the level of Bregma + 0.46 mm 35d after tFCI. *n* = 4–6/group. **l**–**m** Correlation analysis of N1 with forepaw fault rate (**l**) and hind paw fault rate (**m**) 35d after tFCI. *n* = 4–6/group. *p < 0.05, **p < 0.01, ***p < 0.001, ****p < 0.0001, cKO-MCAO vs*.* WT-MCAO; ^#^*p* < 0.05, ^##^*p* < 0.01, ^###^*p* < 0.001, ^####^*p* < 0.0001, WT-MCAO or cKO-MCAO vs. Sham, or as indicated. Multiple *t* tests, one-way ANOVA or two-way ANOVA repeated measurement and Bonferroni post hoc statistical test assessed significance

Our studies revealed SIK3-cKO promoted white matter integrity by regulating Mi/MΦ phagocytosis. Finally, we evaluated whether SIK3-cKO promoted neurological recovery long term after tFCI. SIK3-cKO did not affect sensory and motor functions under normal physiological conditions (sFig. 7a–c). In the first week after tFCI, using a rough assessment method 7-point neurological score, there was not any significant difference between WT and cKO mice (sFig. 7d). We further used a battery of behavioral tests to assess neurological deficits after tFCI. In the rotarod test, deficits in motor coordination were significantly increased by tFCI. Compared to WT mice, SIK3-cKO mice showed a significant improvement in movement time after tFCI (Fig. 7a). In the grid-walking test, which measures locomotor function, SIK3-cKO mice showed a significantly decreased fault ratio of the forepaw and hindpaw compared with WT mice after tFCI (Fig. 7b, c). These results indicated that SIK3-cKO promoted long-term neurological recovery of sensory and motor functions after tFCI.

The formation of currents between two junctional proteins of nodes of Ranvier (NORs) allows excitation to be conducted in a hopping manner, greatly accelerating the conduction of myelinated fibers [43]. We performed Nav1.6/Caspr immunostaining to evaluate the number and morphology of NORs (Fig. 7d, sFig. 7f). tFCI led to NORs shortening and loss, while SIK3-cKO partially preserved the number and length of NORs (Fig. 7e–g). The paranode length was correlated with latency to fall (Fig. 7h). Compound action potentials (CAPs) represent synchronous firing of numerous axons and previous results from our group showed the N1 peak responded to myelinated axonal conduction and the N2 peak responded to unmyelinated axonal conduction [44] (Fig. 7i). SIK3-cKO promoted recovery of the amplitude of N1, but not N2 (Fig. 7j, k, sFig. 7e), suggesting that SIK3-cKO promoted white matter functional integrity by reducing demyelination (N1), not axonal injury (N2). These results were consistent with the immunostaining of MBP. Furthermore, the amplitude of N1 was negatively correlated with the fault ratio of both the forepaw and hindpaw in the grid-walking test (Fig. 7l, m). Together, SIK3-cKO protected myelin integrity, which led to long-term recovery of neurological function.

In summary, our study demonstrated that the pro-inflammatory Mi/MΦ phenotype performed excessive phagocytosis, and the anti-inflammatory Mi/MΦ phenotype performed normal phagocytosis in the tFCI mice model. Using SIK3-cKO transgenic mice, we discovered that SIK3-cKO inhibited Mi/MΦ activation and heterogenized Mi/MΦ from the Transit toward the Anti phenotype. SIK3-cKO regulated Mi/MΦ phagocytosis of neurons indiscriminately, promoting both normal phagocytosis of apoptotic neurons and excessive phagocytosis of live neurons. In contrast, SIK3-cKO selectively regulated Mi/MΦ phagocytosis of myelin by promoting normal phagocytosis of myelin debris, while inhibiting excessive phagocytosis of myelin sheath. The regulation of phagocytosis by SIK3-cKO promoted the recovery of white matter, which persisted long term, ultimately promoting neurological recovery.

## Discussion

Few studies have examined the normal function of SIK3 within the CNS system or its role in CNS diseases [19]. Knockdown of SIK3 has been shown to promote anti-inflammatory effects [18] by regulating Mi/MΦ toward the anti-inflammation phenotype; thus, we examined whether SIK3-cKO would exert similar effects after tFCI and lead to an attenuation of tissue damage and neurological deficits. We found that SIK3-cKO promoted long-term functional recovery and white matter integrity after tFCI but did not improve gray matter integrity. Specifically, SIK3-cKO inhibited Mi/MΦ activation and promoted Mi/MΦ heterogenization toward the anti-inflammatory phenotype, which enhanced the excessive phagocytosis of live neurons and normal phagocytosis of apoptotic neurons after tFCI. However, whereas SIK3-cKO attenuated excessive phagocytosis of myelin sheath after tFCI, it promoted normal phagocytosis of myelin debris. This excessive phagocytic role of Mi/MΦ was positively correlated with the pro-inflammatory phenotype Mi/MΦ.

In previous studies, only the anti-inflammation Mi/MΦ phenotype was shown to act as phagocytes, not pro-inflammation Mi/MΦ [27]. In addition, the general consensus held that these anti-inflammation Mi/MΦ phagocyted only apoptotic cells, toxic substances, and inflammatory cells (e.g., “normal phagocytosis”). However, our scRNA-seq results showed pro-inflammation Mi/MΦ possess phagocytic function and that there were significant differences in the nature of the phagocytic functions between pro-inflammation and anti-inflammation Mi/MΦ, not only in the expression of phagocytosis-related genes, but also in enriched GO terms. Altogether, these results proved that the pro-inflammation Mi/MΦ phenotype performed “excessive phagocytosis,” whereas the anti-inflammation phenotype Mi/MΦ performed “normal phagocytosis.” Interestingly, phagosome and lysosome-related genes were higher in pro-inflammation Mi/MΦ than anti-inflammation Mi/MΦ, suggesting possibly that living cells needed higher phagosomal and lysosomal capacity to be decomposed compared to apoptotic cells. Another interesting phenomenon is that triggering receptor expressed on myeloid cells-2 (TREM2) was highly expressed in the Pro and Transit Mi/MΦ in RNA-seq datasets (Fig. 1h: excessive phagocytosis). Lots of previous studies have proved that TREM2 promotes Anti and Transit heterogeneity [45, 46]. This is a special finding that needs further investigations.

Previous studies have reported that heterogenization of Mi/MΦ toward the anti-inflammation phenotype was accompanied by enhanced phagocytosis of neurons [7] and that inhibiting this shift attenuated phagocytic activity [32]. However, these studies failed to account for the role of Mi/MΦ heterogenization in normal or excessive phagocytosis. In our studies, we observed that SIK3-cKO shifted Mi/MΦ heterogenization from the transitional phenotype to the Anti phenotype after tFCI and upregulated phagocytic activity. Correlation analysis revealed that the Anti phenotype was positively correlated with normal beneficial phagocytosis, though a higher number of live neurons were also phagocyted as assessed with Iba1/NeuN/Caspase3 triple immunostaining. These results were consistent with previous findings from our group with TBI models [29] and indicated that these anti-inflammation Mi/MΦ could not discriminate between apoptosis and live neurons. In contrast to neurons, the Anti Mi/MΦ were quite discriminating when it came to phagocyting damaged myelin versus non-damaged myelin, as the Anti phenotype was positively correlated with myelin integrity. The pro-inflammatory phenotype, on the other hand, was positively correlated with excessive phagocytosis. However, it remained unknown what caused the difference in Mi/MΦ recognition of neurons and myelin. The effects of SIK3-cKO on Mi/MΦ heterogeneity were only observed in STR while not in CTX (Fig. 3d, i). In CTX, SIK3-cKO did not lead to significant downregulation of SIK3 protein and did not affect the phagocytosis of Mi/MΦ toward the cell bodies of neurons 3d after tFCI (sFig. 5d). All of these are consistent with our conclusion that SIK3-cKO has no effect on the gray matter.

Our results are the first to show that a delicate balance exists in the CNS after injury that was mediated by SIK3, such that over representation of the pro-inflammatory phenotype facilitates excessive removal of myelin sheaths that is detrimental to restoration and repair, yet over representation of the Anti Mi/MΦ phenotype promotes white matter integrity. Although SIK3-cKO also promoted the rest Mi/MΦ heterogenization, the nature of this association was unknown as we could not classify the rest Mi/MΦ in our scRNA-seq data 5d after tFCI. We inferred that most of Mi/MΦ were activated and expressed the pro-inflammatory or anti-inflammatory chemokines in 5d after tFCI. Therefore, there exist few rest Mi/MΦ. This may lead to the proportion of rest Mi/MΦ being too low to be classified by Seurat in RNA-seq analysis. This is also consistent with the result of our immunofluorescence staining 3d after tFCI, which indicated that the rest Mi/MΦ only accounts for about 1% of Mi/MΦ (Fig. 3f–k). Thus, more studies are needed to determine the phagocytic function of the rest Mi/MΦ phenotype.

The beneficial role of Mi/MΦ has been extensively studied. Indeed, the anti-inflammation Mi/MΦ phenotype releases anti-inflammatory factors and neurotrophic factors to promote proliferation, differentiation, and maturation of oligodendrocyte precursor cells (OPCs) in white matter-related diseases, such as TBI and multiple sclerosis [7, 47]. It also rapidly clears myelin debris [48]. On the other hand, we found that some Mi/MΦ expressing pro-inflammatory phenotype entered into white matter bundles, which could result in myelin loss via excessive phagocytosis of otherwise non-damaged myelin, which we termed “excessive phagocytosis.” These results suggested that excessive phagocytosis by Mi/MΦ may be discounted as a possible cause of demyelination. Apart from SIK3, studies of our group showed that many other genes could affect the heterogeneity of Mi/MΦ, such as TAK1 and STAT6, thus affect the expression of CD16 in Mi/MΦ [32, 49]. This may explain the discrepancy of CD16^+^ cells and SIK3^+^ cells’ trendencies in Fig. 2d, e in our manuscript. However, we still found SIK3 played an important role in Mi/MΦ heterogeneity in our study. SIK3-cKO promoted normal phagocytosis of myelin debris by Mi/MΦ and inhibited excessive phagocytosis, suggesting that SIK3-cKO may protect white matter by inhibiting demyelination. We do recognize, however, that it is premature to classify the behavior of Mi/MΦ as “normal phagocytosis” or “excessive phagocytosis” based purely on their phenotype, and that additional experiments, such as in vitro experiments, are needed to further prove the relationship between Mi/MΦ heterogenization and normal/excessive phagocytosis.

There were several shortcomings in our study. Firstly, limitations in the imaging depth of two-photon microscopy precluded our ability to use this technology to monitor Mi/MΦ excessive phagocytosis of myelin in STR as it could only scan Mi/MΦ in cortical surfaces, where excessive phagocytosis was not observed. Secondly, we only used NeuN/Caspase3 immunofluorescence staining to distinguish apoptotic/live neurons phagocyted by Mi/MΦ, which could not provide any knowledge as to whether these neurons were functional, as could be discerned from patch clamp analysis. Importantly, we only observed SIK3-cKO regulation of normal phagocytosis and excessive phagocytosis in the acute stage, thus mechanistic information during the chronic phase was lacking. Finally, the mechanism of SIK3 knockout in Mi/MΦ promoting the Anti microglia heterogeneity is still not clear. We preliminarily found that SIK3-cKO regulated the complemental system in Mi/MΦ to promote Mi/MΦ heterogenization into the Anti and Transit phenotype. But this needs further investigations to make the data stronger.

In conclusion, our study identified SIK3 in Mi/MΦ as an important target to protect long-term white matter integrity and enhance functional recovery after tFCI. SIK3-cKO promoted non-specific phagocytosis of neurons as well as specific phagocytosis of myelin sheaths. SIK3-cKO promoted normal phagocytosis of myelin debris by Mi/MΦ and inhibited excessive phagocytosis of myelin sheath that correlated with heterogenization of Mi/MΦ to the Anti phenotype.

## Supplementary Information

Below is the link to the electronic supplementary material.

Supplementary file1 (DOCX 2525 KB)

## Data Availability

All primary data and materials in the manuscript are available upon reasonable request.
