# Supplementary material for: The role of microglial/macrophagic salt-inducible kinase 3 on normal and excessive phagocytosis after transient focal cerebral ischemia
Source: Cell Mol Life Sci. 2022 Jul 21;79(8):439. doi: 10.1007/s00018-022-04465-1 (PMC9304053; doi:10.1007/s00018-022-04465-1)
Supplement: Supplementary file 1 — Supplementary file1 (DOCX 2525 KB) [file 18_2022_4465_MOESM1_ESM.docx]

**Supplementary Materials**

**Methods**

**Animal ethics and experimental groups**

Two transgenic C57BL/6J mice: (i)B6.129P2(Cg)-Cx3cr1tm2.1(Cre/ERT2) Litt/WganJ (The Jackson Laboratory, stock: 021160); (ii) SIK3 Flox^+/+^ (Shanghai Southern Model Biological Corporation construct). The target transgenic mice (SIK3-cKO) were obtained after mating SIK3 ^Flox/flox^ with Cx3cr1^CreER^ mice by tamoxifen-induced for experiments. Cx3cr1^CreER^ knock-out mice express a Cre-ERT2 fusion protein and an enhanced yellow fluorescent protein (EYFP) from endogenous Cx3cr1 promoter/ enhancer elements. EYFP immunofluorescence is observed Cx3cr1-expressing microglia in the brain, mimicking endogenous gene expression patterns. When Cx3cr1^CreER CreER^ are bred with mice containing loxP-flanked sequence, tamoxifen-inducible, Cre-mediated recombination will result in deletion of the floxed sequences.

Mice were kept in a well-ventilated environment with a free and adequate diet and water, with 12 hours of light/12 hours of darkness daily and controlled room temperature at 23-25°C. SIK3-cKO or littermate wild-type male mice (8-12 weeks) were randomly divided into four groups: WT-Sham, cKO-Sham, WT-MCAO, cKO-MCAO. Mice were induced by intraperitoneal injection of tamoxifen to induce specific knockdown of SIK3 on Mi/MΦ at a concentration of 100 mg/kg (20 mg/ml) once daily for 5 days. All animal procedures were approved by the Animal Care and Use Committee of Fudan University and performed in accordance with the National Institutes of Health Guide for the Care and Use of Laboratory Animals. All animal experiments were reported in compliance with the ARRIVE guidelines.

**Murine models of tFCI**

A transient middle cerebral artery occlusion (MCAO) for 60 minutes was used as *Transient Focal Cerebral Ischemia (tFCI),* described previously^47^. A transient left middle cerebral artery occlusion (MCAO) for 60 minutes was used as *Transient Focal Cerebral Ischemia (tFCI),* described previously^47^. Briefly, the external carotid artery (ECA) was ligated and cut. The nylon (diameter 0.10mm) monofilament coated with a silicone tip (0.21- 0.23mm) was inserted into the ECA and advanced along the internal carotid arteries until it meets resistance (occluding the middle cerebral artery). To ensure the success of the tFCI model, we used laser Doppler to monitor the local cerebral blood flow (CBF) during ischemia. If the cerebral blood flow in the middle cortical cerebral artery decreased <75% during cerebral ischemia in mice, it was considered a failure of modeling and the experimental animal would be excluded.

**Single cell RNA-seq analysis**

Single cell RNA-seq raw data (GSM5220257, GSM5220258) were downloaded from GEO website^48^. In this dataset, CD45^high^ cells were collected from ischemic mouse brains 5d after 60 min tFCI. Cells ranging from 200 to 2500 detected genes per cell with less than 5% of genes encoding mitochondria were applied for analysis. Seurat V 4.0.3 was used for downstream analysis, including UMAP, heatmap, feature plot and Dot plot. Differentially expressed genes (DEGs) were defined as whose fold change >2 or <-2, adjusted p-value <0.05 between two groups. Gene ontology (GO) enrichment analysis was performed in g: Profiler and Metascape website. GOplot V 1.0.2 was used for chord plot. Graphpad V 8.2.1 was used for bar plot and volcano plot. Monocle V 2.20.0 was used for pseudotime analysis.

**Sensorimotor outcomes**

Behavioral tests of sensorimotor functions were as described previously^49^. Behavioral tests were performed by an individual blinded to experimental groups. Sensory and motor functions were measured at pre, 3, 5, 7, 14, 21, 28, and 35d after tFCI.

*Neurological Score*

Neurological dysfunction was given a score of 0-7: 0 as no neurological dysfunction; 1 as inability to fully straighten the right front paw; 2 as reduced anterior grasp; 3 as turning in all directions when grasping the tail; 4 as circling or walking only to the right; 5 as walking during stimulation; 6 as unresponsive to stimulation; and 7 as death. Score were assessed at 1, 2, 3, 4, 5, and 7d after tFCI.

*Rotarod test*

Mice were trained 3 days prior to tFCI to learn to run on an accelerating rotating bar at an initial speed of 5 rpm, after which the uniformly accelerating motion was maintained until reaching 40 rpm at 300 seconds, and remained at a uniform speed of 40 rpm for 300-400 seconds. Record the time of mice dropping.

*Adhesive remove test*

Two sticky paper patches (3mm x 4mm) were applied precisely to each forepaw. The time required to remove each sticker was recorded and tested twice daily at 5 min intervals for each forepaw. training was started 3 days prior to tFCI to ensure performance and to limit individual variability.

*Grid-walking test*

The experimental setup was an elevated grid surface (40 L x 20 W x 31 H cm) with a grid opening of 4 cm^2^. Mice were placed on the wire grid 3 days before tFCI to train walking, and 3min video of mice walking on the grid was taken after tFCI to calculate the foot-fault ratio of the forepaw and hind paw on the injured side. Fault rate =fault steps/total steps*100%.

**Primary microglia culture**

Primary microglia culture was prepared as described previously^50^. SIK3-ko microglia were isolated from SIK3 flox^+/+^ Cx3cr1 cre^+/+^ fetal mice. Primary microglia culture was prepared as described previously^1^. SIK3-ko microglia were isolated from SIK3 flox^+/+^ Cx3cr1 cre^+/+^ fetal mice. At P0, fetal transgenic mice were subcutaneously injected with tamoxifen 10ul (20 mg/ml) near the brain with a micro syringe. WT mice were injected with equivalent PBS at P0. The cortical tissue of newborn mice for 3 days was separated into single cell suspension by Trypsin (0.01%) at 37˚C for 15 min. Then, inoculated into a culture bottle coated with polylysine. Microglial cells were washed with ice-cold DMEM containing 5% fetal bovine serum (FBS) to stop digestion. The extracted microglia and the culture medium were in the same culture bottle. After about 10 days of culture with DMEM solution containing 10% FBS, microglia were removed at 200 rpm and 37 ° C for primary microglia culture. The seed plate was centrifuged at 215g for 5 minutes, the supernatant was discarded, a small amount of DMEM was added to resuspend the cells, and the seed plate could be used in the experiment after culture for nearly 3 days.

**Immunofluorescence staining**

Floating coronal brain slices were blocked with 10% serum in 0.3% Triton X-100 in PBS (0.3%PBST) for 1h at room temperature, then overnight incubation with primary antibodies at 4 ˚C refrigerator. After three times washing for 10min in 0.3% PBST, sections were incubated with the appropriate secondary antibodies for 1h at room temperature. Sections were then washed three times for 10min in 0.3% PBST, and mounted with DAPI Fluoromount-G or Fluoromount-G (Southern Biotech; Birmingham, AL, USA). While using the mouse primary antibody, the M.O.M kit (BMK-2202, Vector Laboratories; Burlingame, CA, USA) was applied to block nonspecific signals. Primary antibodies included: SIK3 Rabbit (ab88495, Abcam, 1:200), MBP Rabbit (ab40390, Abcam, 1:1000), MBP Rat (ab7349, Abcam, 1:1000), NF200 Rabbit (ab8135,Abcam,1:1000), NeuN Rabbit (ab177487, Abcam,1:1000), NeuN Mouse (MAB377X, MerckMilipore,1:1000), Iba1 Rabbit (ab178846,Abcam,1:1000), Iba1 Goat (ab5076,Abcam,1:1000), CD16/32 Rat (Cat553142,BD Pharmingen,1:200), CD206 Goat (AF2535,R&D,1:200), CD68 Mouse (ab201340,Abcam,1:200), Caspase3 Rabbit (9661S,CST,1:200), C1q Mouse (ab71940,Abcam,1:100), C3 Rat (ab11862,Abcam,1:100), CD47 Rabbit (orb389308,Biorbyt,1:200), SIRPα Rabbit (ab8120,Abcam,1:500), Nav1.6 Rabbit (ab65166,Abcam,1:500), Caspr (MABN69,Milipore,1:300). Secondary antibodies were all purchased from Jackson ImmunoResearch Inc. (1:1000).

**In vivo analysis of Mi/MΦ phagocyting neuronal cell bodies**

According to the size of area in which Mi/MΦ contacts with neurons, 4 phagocytic types of “Engulf”, “Enwrap”, “Touch” and “No Touch” are determined. Mi/MΦ of “Engulf” phenotype completely phagocyte the neuronal cell body, the morphology of Mi/MΦ showed a large round shape with shorter processes. “Enwrap” Mi/MΦ wrap more than half of neuronal surface, and herein the morphology of Mi/MΦ appears flat and irregular shapes. “Touch” Mi/MΦ contacted neurons through cell bodies or slender processes, but the contact surface area with neurons was less than half of that of neurons, the morphology of Mi/MΦ showed small round shape with longer processes. And “No Touch” don’t contact neuron in space. “Engulf” “Enwrap” and “Touch” types represent the different degree of Mi/MΦ phagocyte neuron (Iba1^+^NeuN^+^), while “No Touch” state doesn’t phagocyte neuron (Iba1^-^NeuN^+^).

**Flow cytometry**

Flow cytometric analysis was performed using a FACS flow cytometer as describe previously to measure SIK3’s expression on different types of cells at 3d after tFCI^51^. Sorting of Mi/MΦ from WT and SIK3-cKO mice by flow cytometry.

Antibodies used in flow cytometric staining were listed as below: CD45-eFluor 450 (48-0451-82, eBioscience, 1:50), CD11b-APC cy7 (47-0112-82, eBioscience, 1:200), O4-PE (130-117-507, Miltenyi Biotec, 1:100), Tubulin Beta3 (TUBB3)-Alexa Fluor® 488 (657404, BioLegend, 1:100), SIK3 (ab88495, Abcam, 1:200).

Data analysis was performed using FlowJo software (version 10.0).

Sorting of Mi/MΦ from WT and SIK3-cKO mice for detecting the SIK3 expression. The mice were anesthetized with chloral hydrate and HBSS was perfused rapidly. The brain tissue was separated and put into the digestive tube, and the digestive tube was connected to the digester. Brain tissue homogenate was filtered through a 70µm filter and transferred to a precoll tube. Mix well to make the whole homogenate 30% percoll. Slowly and carefully inject 70% percoll into the bottom of the tube containing 30% percoll homogenate. After centrifugation at 800 rcf, 18℃ for 30 minutes, a clear plane line should be seen at the junction of 70%-30% percoll. A pipette was used to collect 70%-30% of the interfacial flocs into a new tube and centrifuged at 800 rcf and 18℃ for 10 minutes. The cells were blocked with mouse CD16/32 for 10 minutes, then stained with CD45-eFluor 450, CD11b-APC cy7, CD11c-PerCP cy5.5 and Ly6G(Gr1)-PE for 30 minutes. After dyeing, HBSS and FBS was added for washing. Ly6G-CD11c-CD11b+CD45^Int^microglia were sorted by the FACS flow cytometer. Antibodies used in flow cytometric staining were listed as below:CD16/32 (16-0161-86, eBioscience, 1:100), CD45-eFluor 450 (48-0451-82, eBioscience, 1:50), CD11b-APC cy7 (47-0112-82, eBioscience, 1:200), CD11c-PerCP cy5.5(45-0114-82, eBioscience, 1:100), Ly6G(Gr1)-PE (12-9669-82, eBioscience, 1:200).

**Real-time Quantitative PCR (qPCR)**

To Identify the knockout effect of SIK3 in microglia, the mRNA expression of SIK3 in sorted microglia by qPCR detection. Total RNA was isolated from mouse brains with the TRIeasy^TM^ Total RNA Extraction Reagent kit (10606ES60, Yeasen). RNA was used to synthesize the first strand of cDNA (11752, Invitrogen). Program for transcription was 65°C for 5 minutes, 25°C for 10 minutes, 50°C for 30 minutes, 65°C for 5 minutes, and 4°C for maintenance. qPCR was performed by using corresponding primers and Hieff^®^ qPCR SYBR^®^ Green Master Mix (11201ES03/08/60, Yeasen). The program for qPCR was 95°C 5 minutes, (95°C 10 seconds, 60°C 30s) × 40 times. Add a melting curve from 50°C–92°C, read every 0.2°C, hold 2s, incubate at 8°C. The results were normalized to GAPDH in each sample as a control. MRNA’s expression were reported as fold change vs. WT-Sham group. The following primers were used for mouse samples: SIK3: TTCCGGGAGGTTCAGATAA (forward); CGTATTCTGTCACCAGGTAAAT (reverse). GAPDH: CTGCCCAGAACATCATCCCT (forward); TGAAGTCGCAGGAGACAACC (reverse).

**Measurements of CAP**

Measurement of *Compound action potential (CAP*) was as described previously^52^. CAPs were measured at 35d after tFCI in CC. The mice were anesthetized and perfused with NMDG cutting solution saturated with a carbon-oxygen mixture (95% O_2_ + 5% CO_2_), and then the brains were quickly severed and removed. The brain slices were transferred to artificial cerebrospinal fluid artificial cerebrospinal fluid(aCSF) and incubated at 32°C for 0.5 h. The slices were incubated at room temperature for 1h and then used for recording.

Concentric circular electrodes were used to stimulate evoked potentials, and 4-7 MΩ glass microelectrodes perfused with aCSF were used for recording. Afterwards, data were acquired using an Axon 1440A digital acquisition system and recorded in current clamp I=0 mode. The stimulating electrodes were placed to CC at about 0.9 mm lateral to the midline, and the recording electrodes were placed at 0.75mm spacing from the stimulating electrodes in CC. CAPs were recorded by sequentially administering 0mA, 250mA, 500mA, 750mA, 1000mA, 1250mA, 1500mA, 1750mA, and 2000mA square wave stimulation.

NMDG solution for brain slices incubation: 17.94g NMDG, 6.5ml HCl, 0.144g NaH_2_PO_4_, 2.52g NaHCO_3_, 4.76g HEPES, 0.19g KCl, 0.99g Na-ascorbate, 0.33g Na-pyruvate, D-glucose 4.955g, Thicurea 0.15g, 1M CaCl_2_ 0.5ml, MgSO_4_.H2O 2.46g, fixed volume to 1000ml with ultrapure water and adjusted the pH to 7.3-7.4.

aCSF solution: NaCl 6.954g, KCl 0.171g, NaHCO_3_ 2.202g, D-glucose 2.180g, 1M CaCl_2_ 0.5ml, 1M NaH_2_PO_4_ 1ml, 1M MgSO_4_ 1.3ml, fixed volume with ultrapure water to 1000ml, adjust the pH to 7.3-7.4.

**Infarct volume measurements**

Measurements of infarct volume and atrophy volume were as described previously^53^. Brain tissue 3d or 35d after tFCI were taken via PA perfusion, and frozen sections were performed starting at the level of the corpus callosum union, and one brain slice of 25 µm was collected every 11 brain slices, for a total of 10 slices collected to form a set. The sets of slices were stained with NeuN immunofluorescence, followed by scanning large images under a fluorescence microscope with a 10x lens for analysis. Volume of infarct or atrophy was determined with NIH ImageJ analysis. Brain infarct or atrophy volume (mm^3^) = Σ (contralateral hemisphere area-noninfarcted volume of the ipsilateral hemisphere area) × 0.3.

**In vitro analysis of microglial phagocytosis**

For microglial phagocytosis of myelin debris, mouse myelin debris was prepared as previously described^54^ and stored in HBSS at −80 °C. 4h after LPS treatment, myelin debris was dissolved in DMEM solution containing 10% FBS by vortexing and sonicating to a final concentration of 1 mg/ml. Four hours later (8h after LPS treatment), took out cultured microglia and fixed in 4% PA. Then perform Iba1/MBP immunofluorescence staining.

For microglial phagocytosis of latex beads, 4h after LPS treatment, latex beads were added with 10 times the number of microglia in DMEM solution containing 10% FBS. Images and films of microglia were photographed by Cytosmart Lux3 cell imaging systems.

**Immunofluorescence and image analysis**

Images were acquired using a fluorescence microscope (20x) or a laser confocal microscope (60x). For brain slices in ipsilateral brain, a field of view 0-800 µm from the damage edge was selected for each brain slice to be considered to be penumbra area. Each slice was taken 2 regions of interest (ROI) in the striatum, 1 ROI in the cortex or corpus callosum; a field of view 800-1200 µm from the damage edge was considered to be core area, each slice was taken 1 ROI in the striatum or corpus callosum. In contralateral hemisphere, each slice was taken 1 ROI in the striatum cortex, or corpus callosum. The average intensity, area or cell numbers of total ROI in the different brain areas were measured for each mouse for statistical.

**Statistical analysis**

All experimental data analysis was performed by double-blind method, and all data are expressed as Mean ± SEM. Differences between WT-Sham, cKO-Sham, WT-MCAO, and cKO-MCAO groups were analyzed using ANOVA and post hoc Student Newman-Keul tests. Immunofluorescence staining was statistically analyzed by FIJI Image J 2.0.0, and electrophysiological CAPs were analyzed by Clampfit 8.2.1. Group comparisons were performed by ANOVA and post hoc Bonferroni tests, multiple t tests to compare single-factor two-group differences, One-way ANOVA to compare single-factor multi-group differences, and Two-way ANOVA to compare two-factor group differences. Graphpad Prism 8.2.1 and Photoshop CS6 were used for plotting. All statistical results p<0.05 were considered statistically different.

**Supplementary Figures**


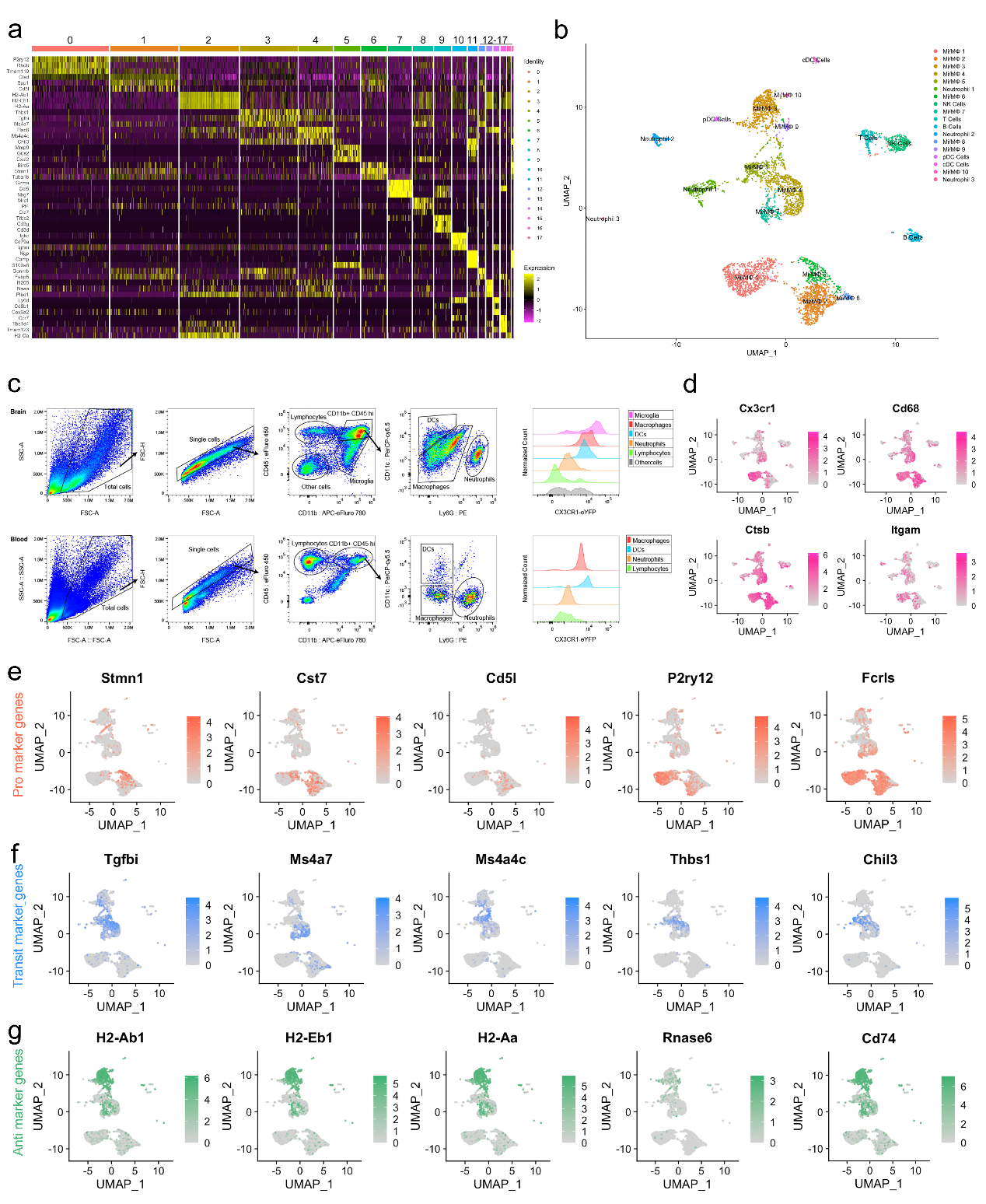


**Supplementary Figure 1. Marker genes of Mi/MΦ by scRNA-­seq.** (a) Heatmap showing the top 3 marker genes for each cluster. (b) UMAP plot showing clusters and cluster annotations of brain CD45^high^ cells 5d after tFCI. (c) The expression level of Cx3cr1 in the immunocytes of the brain and blood in Cx3cr1-eYFP mice 3d after tFCI. (d) Feature plots showing marker genes of Mi/MΦ in CD45 high cells. (e-g) Feature plots showing marker genes of the pro-inflammatory (Pro), anti-inflammatory (Anti) and transitional (Transit) Mi/MΦ.


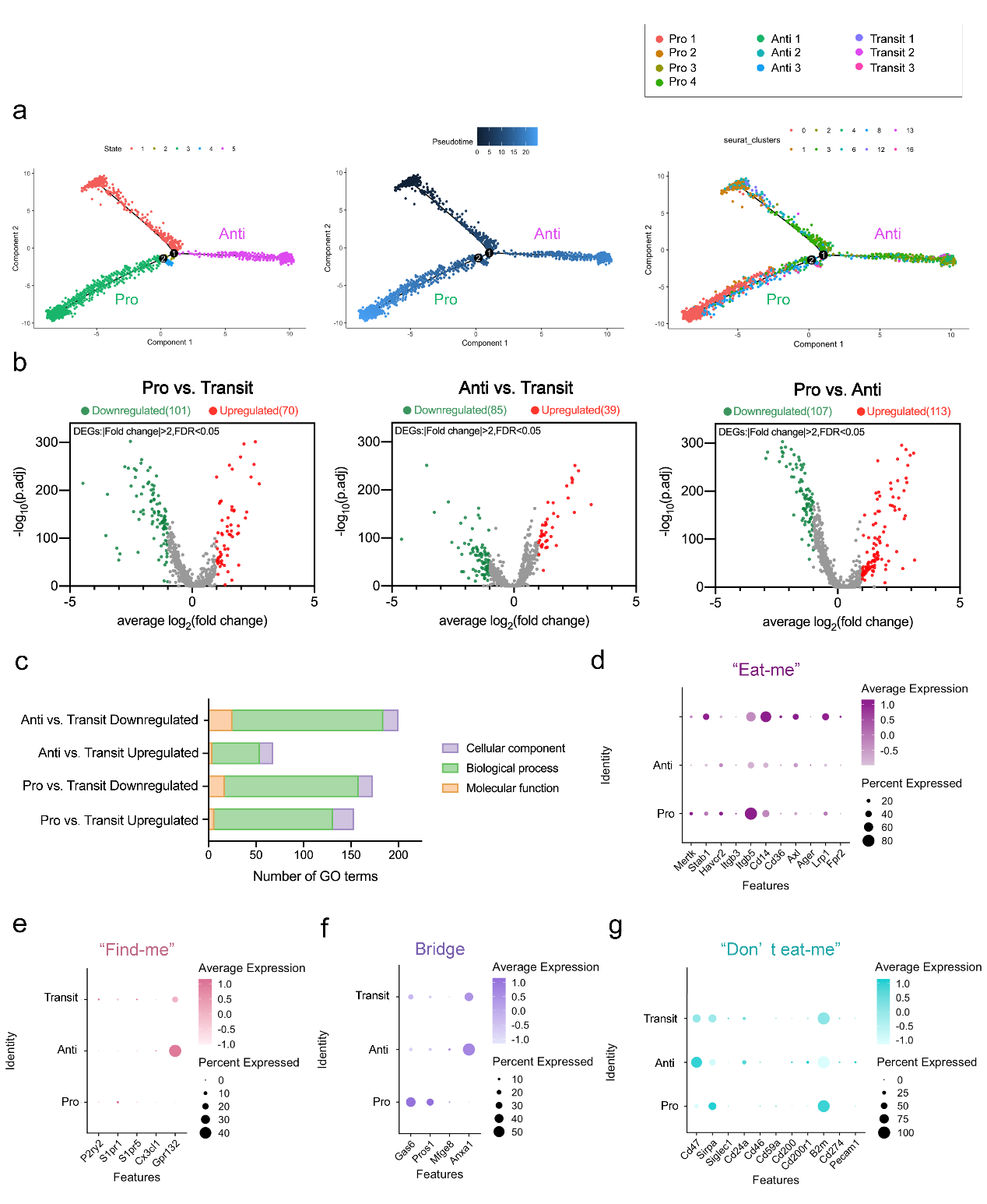


**Supplementary Figure 2. Marker genes of Mi/MΦ by scRNA-seq.** (a) The trajectory analysis of Mi/MΦ in states, pseudotime and clusters by Monocle. (b) Volcano plots showing the DEGs (fold change >2 or <-2, adjusted p value<0.05) in Mi/MΦ. Left panel: DEGs from the Pro Mi/MΦ versus the Transit Mi/MΦ; Middle panel: DEGs from the Anti Mi/MΦ versus the Transit Mi/MΦ; Right panel: DEGs from the Pro Mi/MΦ versus the Anti Mi/MΦ. (c) The numbers of gene ontology (GO) terms in the three categories: Biological process (BP), cellular component (CC), and molecular function (MF). (d-g) Bubble matrix of “eat-me” (d), “find-me” (e), “bridge” (f) and “don’t eat-me” (g) related genes in the Pro, Anti and Transit phenotypic Mi/MΦ.


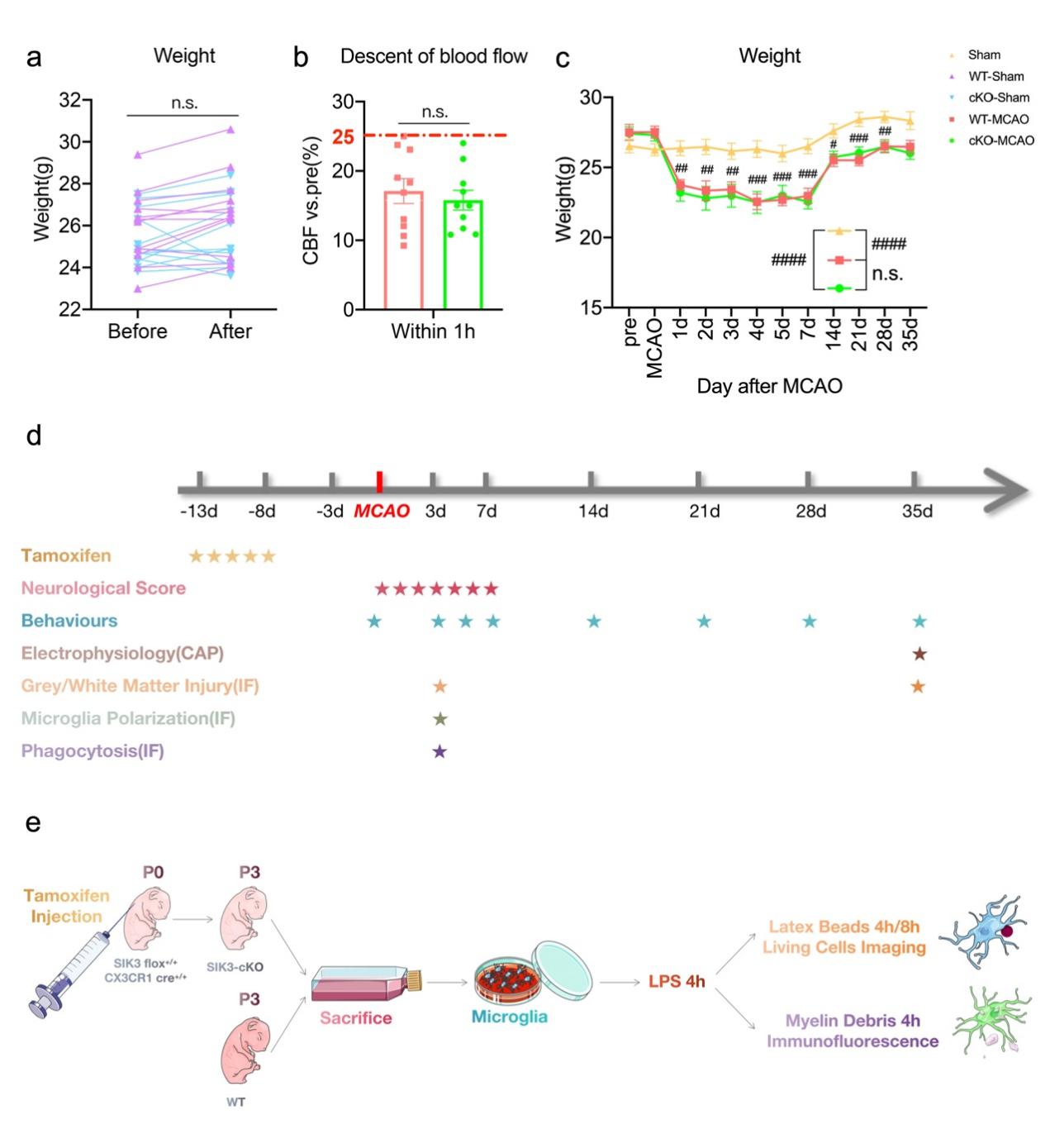


**Supplementary Figure 3. SIK3-cKO didn’t cause cerebral blood flow and body weight change.** (a) Body weight change in WT and cKO mice before and after Tamoxifen injection. (b) The decreasing of CBF in WT-MCAO and cKO-MCAO mice during tFCI. (c) Body weight in Sham (including WT-sham and cKO-sham), WT-MCAO, and cKO-MCAO groups. n=10-12/group. (d) Experimental design of in vivo studies. (e) Experimental design of in vitro studies. **p*<0.05, ***p*<0.01, ****p*<0.001, *****p*<0.0001, as indicated. #*p*<0.05, ##*p*<0.01, ###*p*<0.001, ####*p*<0.001, vs. Sham group, or as indicated. Two-way ANOVA repeated measurement or Multiple t tests and Bonferroni post hoc.


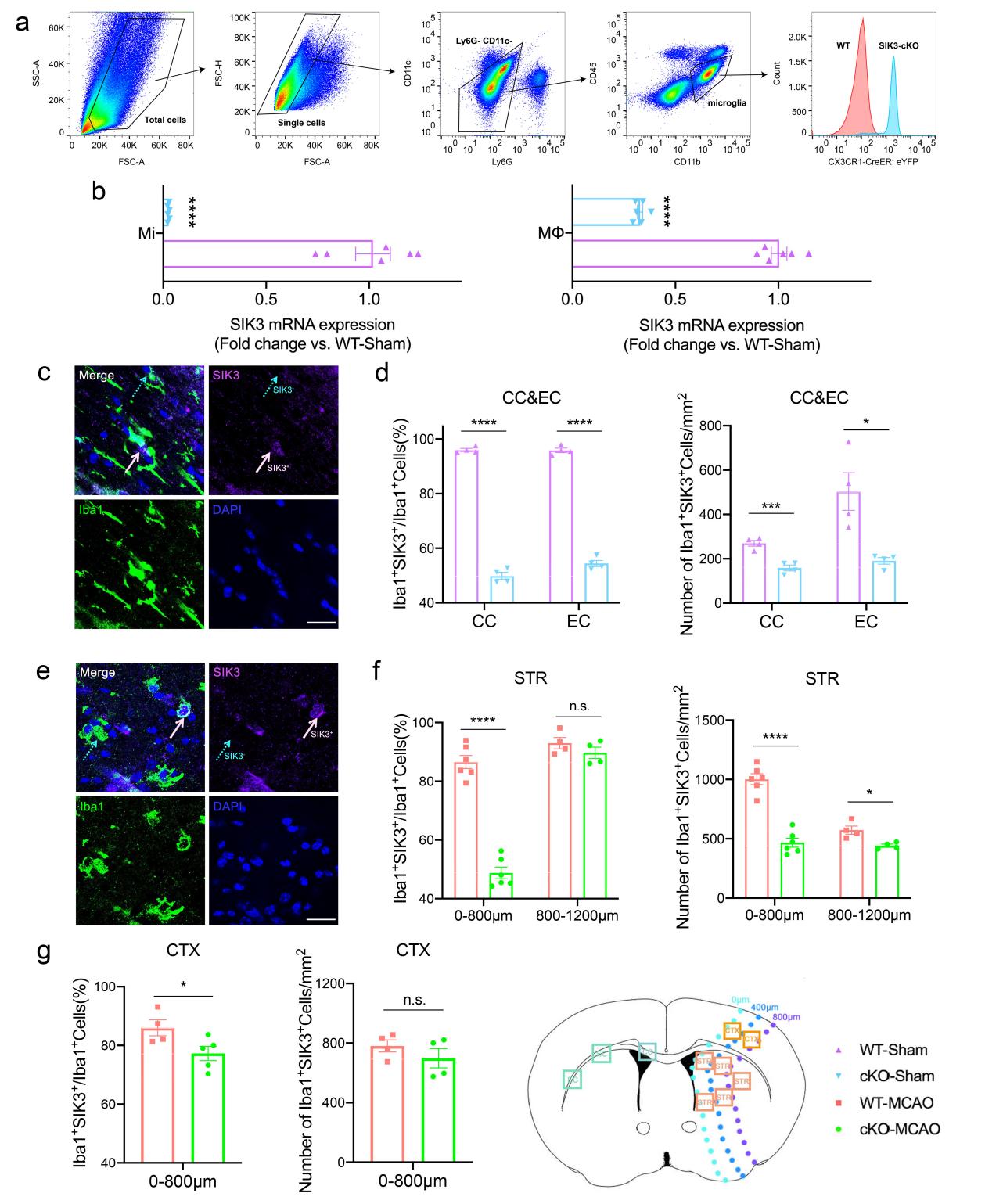


**Supplementary Figure 4. SIK3^+^Mi/MΦ was decreased in transgenic mice.** (a) The CD11b^+^CD45^+^ cell populations were sorted from sham brain by Flow cytometry. (b) mRNA expression levels of SIK3 in Mi/MΦ were measured by real-time PCR (qPCR) in sham groups. (c) Representative images of Iba1/SIK3 staining in the contralateral hemisphere of EC (Scale bar: 20µm). (d) Quantification of percentage of SIK3^+^ microglia in the contralateral hemisphere of CC or EC. (e) Representative images of Iba1/SIK3 staining in STR 3d after tFCI (Scale bar: 20µm). (f) Quantification of percentage of SIK3^+^ Mi/MΦ in 0-800 µm and 800-1200 µm from damage edge in STR 3d after tFCI. (g) Quantification of the percentage of SIK3^+^ in 0-800 µm from damage edge in CTX 3d after tFCI. n=4-6/group. **p*<0.05, ***p*<0.01, ****p*<0.001, *****p*<0.0001, as indicated. #*p*<0.05, ##*p*<0.01, ###*p*<0.001, ####*p*<0.001, vs. Sham group, or as indicated. Mutiple t tests and Bonferroni post hoc.


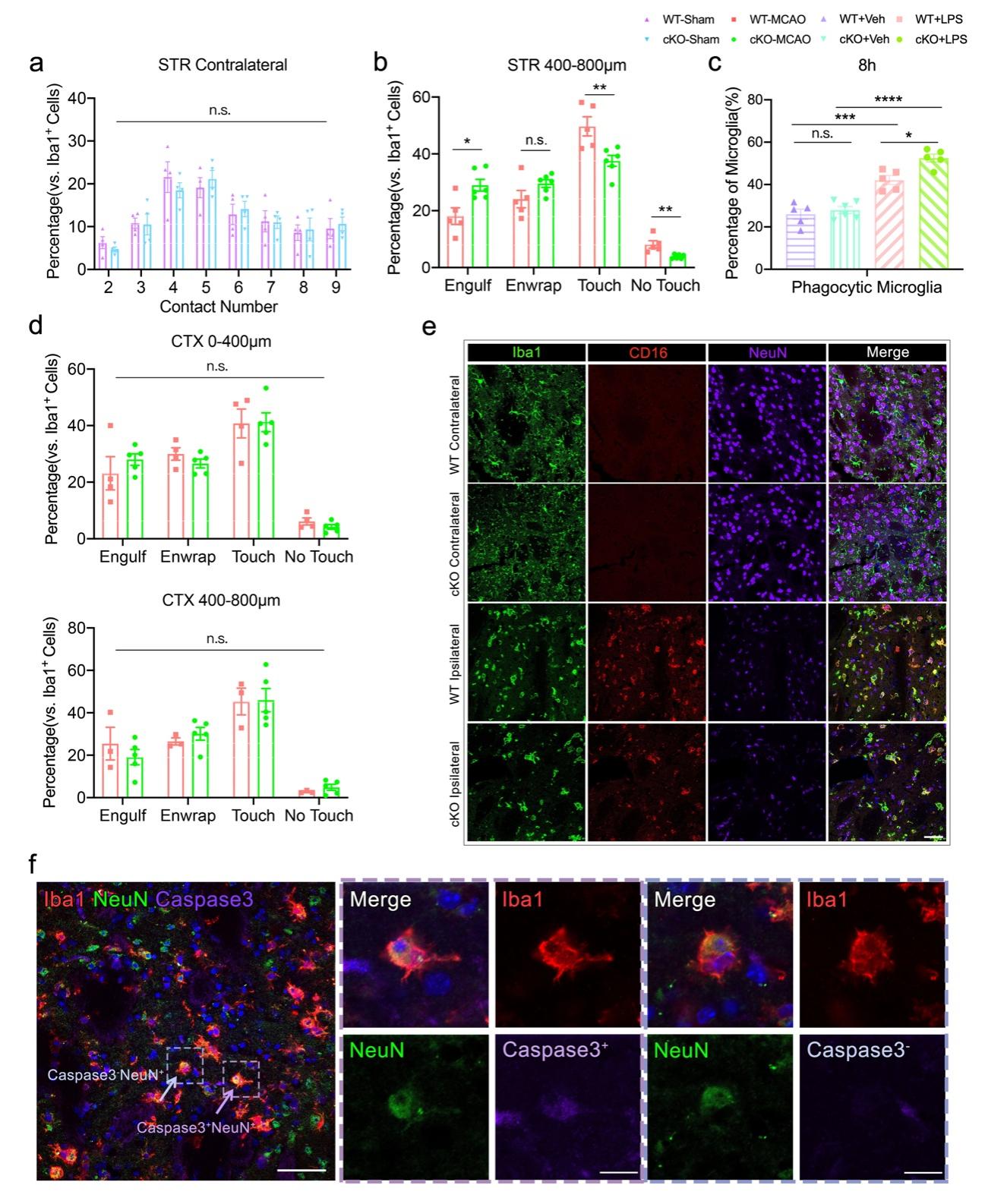


**Supplemental Figure 5. SIK3-cKO regulated Mi/MΦ phagocytosis of neurons after tFCI.** (a) Quantification of the percentage of Mi/MΦ that made contact with neurons grouped by the number of contacted neurons in the contralateral hemisphere of the STR 3d after tFCI. (b) Quantification of the percentage of Mi/MΦ in the “Engulf”, “Enwrap”, “Touch”, and “No Touch” states of phagocytosis in the penumbra within 400-800 µm from the damaged edge in STR 3d after tFCI. (c) Quantification of the percentage of phagocytic Mi/MΦ 8h after adding latex beads (12h after LPS treatment). (d) Quantifications of the percentage of “Engulf”, “Enwrap”, “Touch” and “No Touch” phagocytic types of Mi/MΦ in the penumbra within 0-400µm and 400-800µm from the damage edge in CTX 3d after tFCI. n=3-5/group. (e) Representative images of Iba1/CD16/NeuN immunostaining in the penumbra of STR 3d after tFCI (Scale bar:50µm). (f) Representative images of Iba1/NeuN/Caspase3 in the penumbra (showed in 0-400µm from the infarct border) of STR at 3d after tFCI, with phagocytosis of apoptotic neurons in purple dashed box and phagocytosis of live neurons in the blue dashed box (Left panel, Scale bar: 50µm; Middle panel and Right panel, Scale bar:10µm). ns: no significant, as indicated. Mutiple t tests.


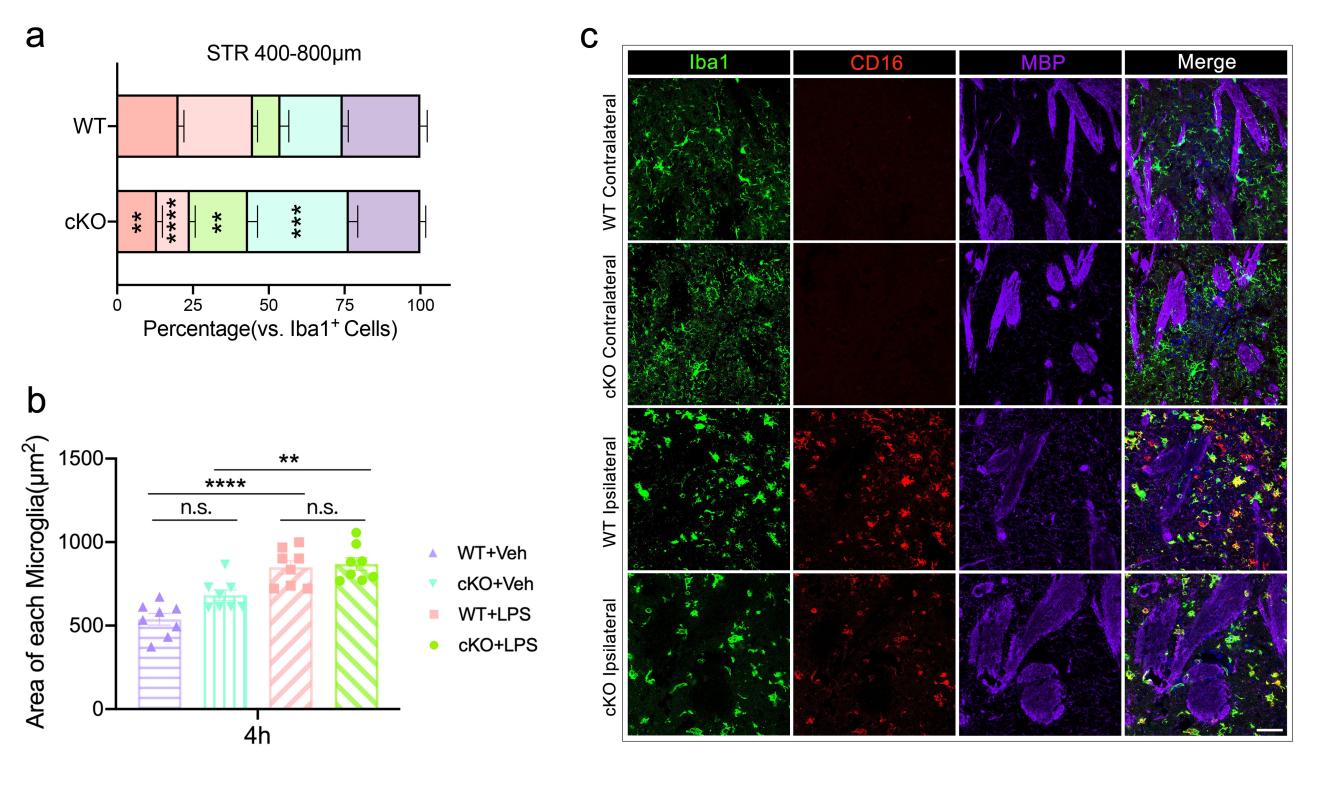


**Supplemental Figure 6. SIK3-cKO regulated CD16^+^Mi/MΦ in different phagocytic states.** (a) Quantification of the percentage of “Internal phagocytosis”, “External phagocytosis”, “Enwrap”, “Touch”, and “No Touch” phagocytic states of Mi/MΦ in the penumbra of the STR (400-800 µm from the damaged edge) 3d after tFCI. (b) Quantification of the area of each microglia 4h after adding myelin debris (8h after LPS treatment). n=8/group. (c) Representative images of Iba1/CD16/MBP immunostaining in the penumbra of STR at 3d after tFCI (Scale bar: 50µm). ***p* < 0.01, *****p* < 0.0001. ns: no significant, as indicated. One-way ANOVA was used to assess statistical analysis.

**
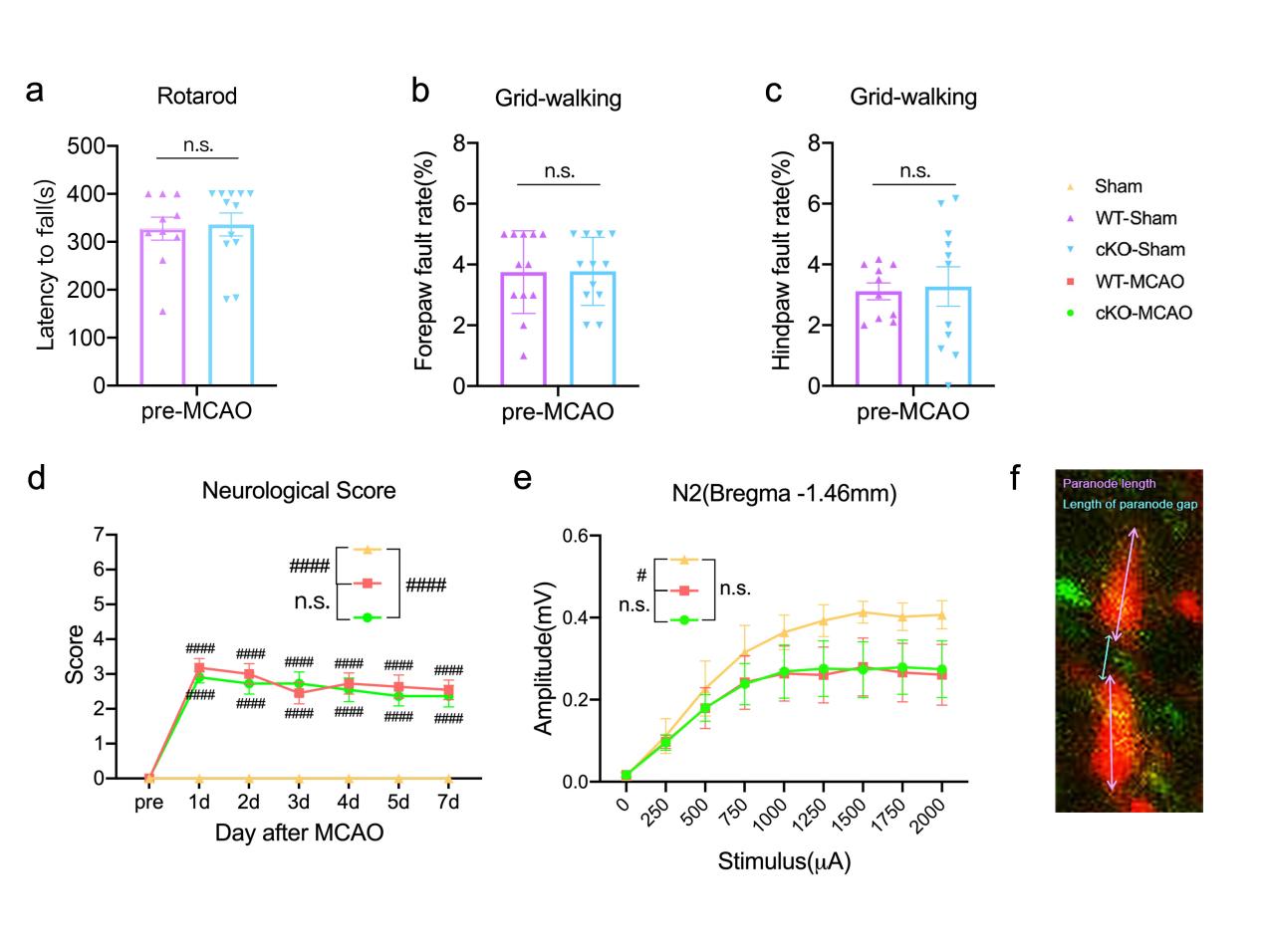
**

**Supplemental Figure 7. SIK3-cKO didn’t improve sensory and motor functions in physiological state.** (a-c) Sensorimotor deficits were evaluated with Rotarod (a) and Adhesive Remove(b-c) tests 1d before tFCI. n=10-12/group. (d) Neurological score was assessed during the first 7d after tFCI. (e) The amplitude of N2 at the level of Bregma -1.46 mm 35d after tFCI. (f) Representative images of a Ranvier node 35d after tFCI. n.s.: no significant, as indicated. Two-way ANOVA or Mutiple t tests and Bonferroni post hoc.
